# Ranking influential nodes in complex networks with community structure

**DOI:** 10.1371/journal.pone.0273610

**Published:** 2022-08-29

**Authors:** Stephany Rajeh, Hocine Cherifi

**Affiliations:** Laboratoire d’Informatique de Bourgogne, University of Burgundy, Dijon, France; University of Alicante, SPAIN

## Abstract

Quantifying a node’s importance is decisive for developing efficient strategies to curb or accelerate any spreading phenomena. Centrality measures are well-known methods used to quantify the influence of nodes by extracting information from the network’s structure. The pitfall of these measures is to pinpoint nodes located in the vicinity of each other, saturating their shared zone of influence. In this paper, we propose a ranking strategy exploiting the ubiquity of the community structure in real-world networks. The proposed community-aware ranking strategy naturally selects a set of distant spreaders with the most significant influence in the networks. One can use it with any centrality measure. We investigate its effectiveness using real-world and synthetic networks with controlled parameters in a Susceptible-Infected-Recovered (SIR) diffusion model scenario. Experimental results indicate the superiority of the proposed ranking strategy over all its counterparts agnostic about the community structure. Additionally, results show that it performs better in networks with a strong community structure and a high number of communities of heterogeneous sizes.

## Introduction

Many real-world systems, including transportation, social, technological, infrastructural, information, and biological systems, are complex. One can represent them using networks where nodes represent their constituent entities and links account for their interactions. Influential nodes in these systems play a critical role in the structure and dynamics of the network [1]. Identifying the most influential nodes in these networks is a major issue. Indeed, it allows conducting specific optimization tasks, such as controlling, minimizing, or maximizing a diffusion process. This issue is mainly related to centrality measures [2]. These measures extract diverse information from the network to quantify its importance. For instance, one can link a node’s capacity to infect others with its Degree and Coreness centrality [3]. Betweenness centrality allows for identifying genes related to heart attacks [4]. Other applications include hindering epidemic outbreaks [5], augmenting the effectiveness of marketing campaigns on social media [6], enhancing the resiliency of infrastructural networks [7], and many other [8–10].

Classically, one can divide centrality measures into local or global measures [2]. Local measures such as Degree and Maximum Neighborhood Component quantify the node’s importance based on its neighborhood. Global measures such as Betweenness and PageRank relate the node influence to its position in the entire network. One can also consider multidimensional measures simultaneously combining local and global information [11, 12]. More recent works exploit the network’s community structure to identify influential nodes [13–20]. They show that the community structure is a crucial factor in effectively quantifying the node’s influence [21, 22]. Centrality measures can also be time-dependent [23, 24].

Centrality measures can be signed (positive or negative) or unsigned (positive and negative). In the first case, one ranks nodes in descending order, and a fraction of the top nodes are selected to conduct a specific optimization task. In the second case, one can have a multitude of ranking schemes. One can use a combination of positive and negative ranks, take a fraction of both positions, or convert negative values to positive values and take the aggregate ranks. In either case, one can have two general ranking schemes, strong and weak. In the former, one selects the most critical nodes first. In the latter, one chooses the less important nodes [25]. Although centrality measures provide an effective way of ranking nodes, several challenges exist. The first challenge is that several centrality measures may underestimate the influence of specific nodes depending on the network’s structure. The second challenge is that many nodes with high centrality may be neighbors. Thus, targeting these nodes for diffusion or immunization is inefficient because one uses the resources locally, ignoring large parts of the network. The third challenge is which ranking schemes and/or combination criteria are ideal for a network with specific topological features.

To address these challenges, we propose a community-aware ranking method. Indeed, communities are pervasive in real-world networks [26–28]. A community is a densely connected and cohesive subgroup of nodes sharing few connections with nodes outside their group. The community structure of a network affects its underlying dynamics [29, 30]. Moreover, ongoing research emphasizes the benefits of the community structure as a basis for identifying influential nodes [13–20]. The proposed ranking strategy exploits this precious information. It is simple yet effective and applicable to all centrality measures. The most straightforward ranking strategy, given a centrality measure, targets the top nodes independently of the community structure if any. Instead, we propose to rank the nodes based on their importance in their communities. First, we select the most central nodes in each community. We order these nodes in decreasing order of their community size. Then we move to the next most central node in each community and adopt the same ordering strategy. We iterate this process until we reach the given budget of nodes to rank. This approach naturally selects distant nodes in each community.

To evaluate the proposed ranking strategy, we report a series of experiments on synthetic and real-world networks using a set of six classical centrality measures using the SIR epidemic model [31]. We categorize these centrality measures into three groups, namely neighborhood-based (Degree and Maximum Neighborhood Component), path-based (Betweenness and Closeness), and iterative refinement-based (Katz and PageRank). Experiments on synthetic networks investigate the impact of various network parameters on the proposed ranking strategy. Indeed, one can control the community structure strength, the community size distribution, and the degree distribution. Real-world networks include infrastructural, social, biological, citation, word, and collaboration networks with unknown community structures. Therefore, we uncover the communities using two community detection algorithms to assess the proposed strategy’s consistency linked to community structure variations. Results show that the community-aware ranking strategy is more effective than the classical ranking by descending order of the centrality measure. The main advantages of the proposed method are threefold:

1. It applies to all types of centrality measures in all types of networks (undirected/directed and unweighted/weighted).
2. It naturally selects distant nodes to expand any diffusion phenomena based on any given budget.
3. Its complexity depends on the centrality measure computed.

## Proposed ranking strategy

Centrality measures aim at quantifying nodes’ influence. Nodes are usually ranked based on the descending order of their influence. Indeed, nodes with the highest centrality are supposed to be strategically located in the network. Thus, targeting them for any optimization task will yield desired outcomes. However, in real-world networks, these nodes may not be far apart, which can be detrimental to the effectiveness of dissemination strategies. Consider an immunization scenario in an epidemic process. Immunizing neighbors in priority, even if influential, may prevent the protection of vast areas of the network. To overcome this issue, we propose a simple ranking method considering the network’s community structure.

### Algorithm

The proposed ranking strategy targets influential nodes spreading across communities in the network. It applies to any centrality measure. First, one computes the centrality of the nodes. Second, one targets top nodes community by community. Such a strategy prevents the concentration of influential nodes in the same network area. The targeted nodes are naturally more dispersed.

**Algorithm 1** Community-aware ranking scheme

**Input**: Graph *G*(*V*, *E*), Centrality measure *β*, Sorted community set *C*, Budget *B*

**Output**: List of ranked nodes *L*

1: *D* ← ⌀      ▹ Compute the centrality of each node

2: **for each** *i* ∈ *V* **do**

3:  *D*[*i*] ← *β*(*i*)

4: **end for**

5: **for each** *c*_*l*,*l*∈{1,2,…,|*C*|}_ ∈ *C* **do**    ▹ Sorting the nodes inside their communities

6:  **for each** *i* ∈ *c*_*l*_ **do**

7:   $D_{c_{l}}\leftarrow D\left[i\right]$

8:  **end for**

9:  $D_{c_{l}}\leftarrow Sort\left(D_{c_{l}}\right)$

10: **end for**

11: **while** *B* ≠ 0 **do**    ▹ Extract sorted list of nodes till budget is reached

12:   **for each** $D_{c_{l}}$ and $i\in D_{c_{l}}$ **do**

13:    **if** $D_{c_{l}}\neq ⌀$ **then**

14:     $v\leftarrow D_{c_{l}}.pop\left(i\right)$

15:     *L*.*append*(*v*)

16:     *B* ← *B* − 1

17:    **end if**

18:   **end for**

19: **end while**

Doing so, it is more likely that any diffusion process spreads more uniformly in the network than in the case where targeted nodes by a centrality measure in a descending order ranking scheme are close to each other. Note that we assume the set of communities is sorted from the biggest to the smallest, with ties decided at random. Also, note that the maximum budget is the size of the network. The pseudocode is provided in Algorithm 1, and a Python version of the code is available on GitHub https://github.com/StephanyRajeh/CommunityAwareRankingScheme.

### Toy example

**Fig 1:**
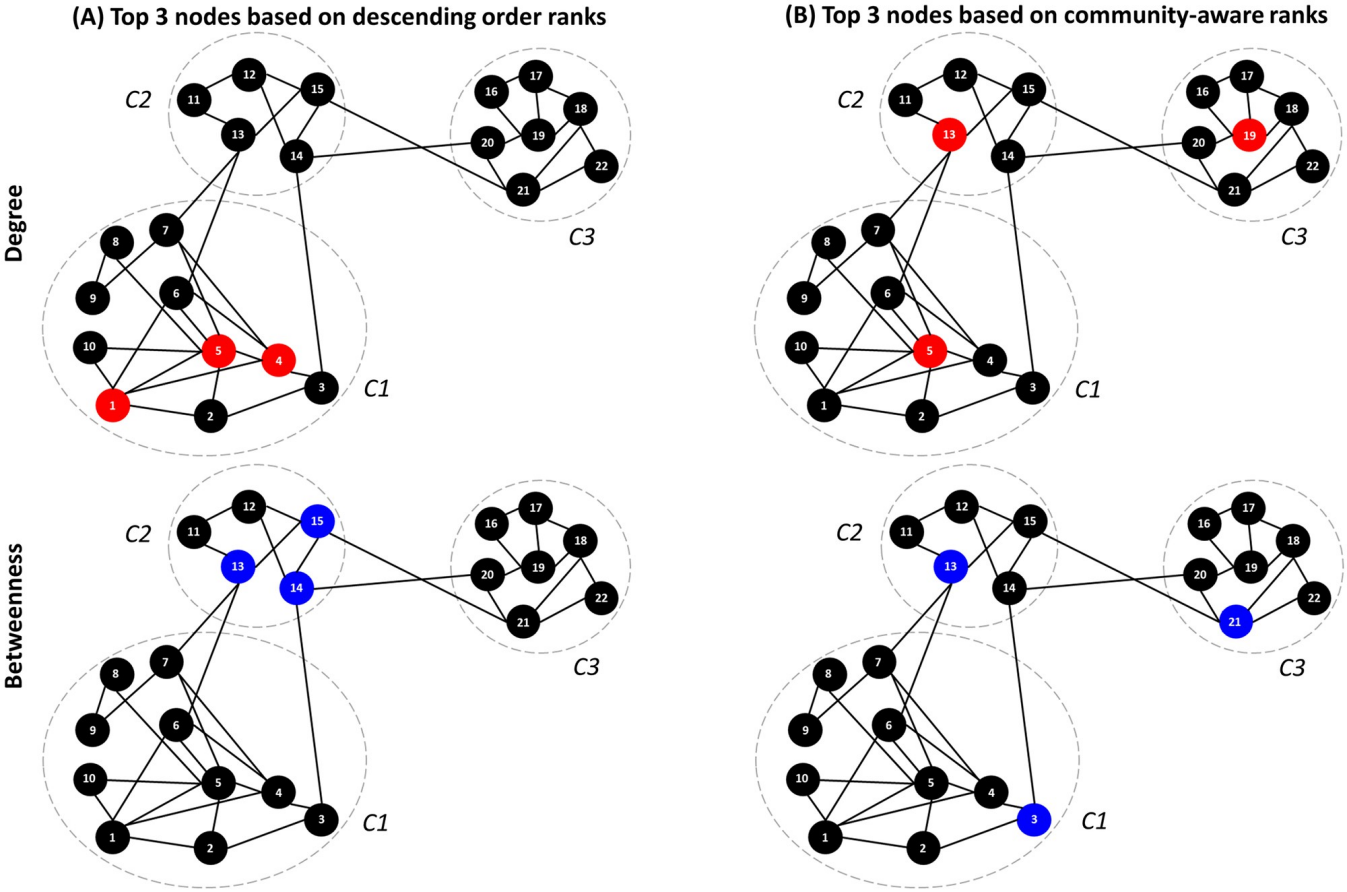
Illustrating the behavior of the descending order ranking scheme and the community-aware ranking scheme. The nodes chosen are the top 3 nodes based on the Degree centrality (colored in red) and the Betweenness centrality (colored in blue).

Fig 1 illustrates the proposed ranking method on a toy example. The network contains 22 nodes and three communities in this example. Suppose the maximum budget is three nodes out of the whole network. We consider Degree and Betweenness centrality as measures of influence. Tables 1 and 2 in S1 Text report the centrality values and the corresponding ranks using the descending order and the proposed approach. Based on the descending order ranking scheme (Fig 1A) of the Degree centrality, we can see that the highest degree nodes (nodes 1, 4, and 5) belong to the same community *C1*. Similarly, the nodes with the highest Betweenness centrality (nodes 13, 14, and 15) are all located in the same community *C2*.

In contrast, the proposed community-aware ranking scheme (Fig 1B) selects the highest degree node in each community. Indeed, node 5 is picked from community *C1*, node 13 is picked from community *C2*, and node 19 is picked from community *C3*. Results with the Betweenness centrality are in the same vein. Instead of targeting the top three nodes in the same community *C2*, the proposed ranking approach selects nodes with the highest Betweenness centrality in each community. More precisely node 3 in *C1*, node 13 in *C2*, and node 21 in *C3*. Note that ranks of nodes with the same centrality value in a community are chosen randomly.

One of the main drawbacks of the classical descending order ranking scheme is ignoring the network’s community structure. From the diffusion perspective, if targeted nodes diffusing a piece of information or a virus are very close, the diffusion dies out before spreading across the other communities. On the contrary, the proposed ranking approach naturally selects the most influential nodes in their community. Indeed, the proposed ranking scheme favors nodes from all the dense parts of the network rather than specific communities.

## Synthetic networks

We investigate synthetic networks using the LFR benchmark [32]. It allows generating modular networks with controlled power-law degree (*γ*) and community size (*θ*) distributions. In addition, one can also tune the community structure strength through the so-called mixing parameter (*μ*). Small values of *μ* indicate a strong community structure with few links between communities. Weak community structures correspond to high values of *μ* with a high fraction of connections between communities. Although one cannot tune the transitivity [33], the LFR benchmark assures generating networks with realistic features [34]. We perform a comparative evaluation of the community-aware ranking method with the classical descending order ranking method based on the SIR diffusion model. Simulation involves a set of synthetic networks with diverse values for the mixing parameter (*μ*), community size distribution (*θ*), and degree distribution (*γ*). Table 1 reports these parameters values.

**Table 1:** Synthetic networks’ parameters generated by the LFR model.

Network parameter	Value
Number of nodes	2500
Average degree	8
Maximum degree	27
Exponent for community size distribution (*θ*)	[2, 2.7, 3]
Exponent for degree distribution (*γ*)	[2, 2.7, 3]
Minimum community size	4
Maximum community size	250
Mixing parameter (*μ*)	[0.05, 0.10, 0.20, 0.40, 0,70]

### Influence of the community structure strength

This experiment aims to investigate the influence of the community structure strength on the performance of the ranking strategies (the descending order ranking scheme and the proposed community-aware ranking scheme). The mixing parameter (*μ*) is tuned to cover a wide range of community structure strengths. It spans from very strong to very weak (*μ* = 0.05, 0.10, 0.20, 0.40, 0.70). Remember that a low value means few links between communities, indicating a strong community structure. In contrast, high value corresponds to networks with many links between communities, indicating a weak community structure.

**Fig 2:**
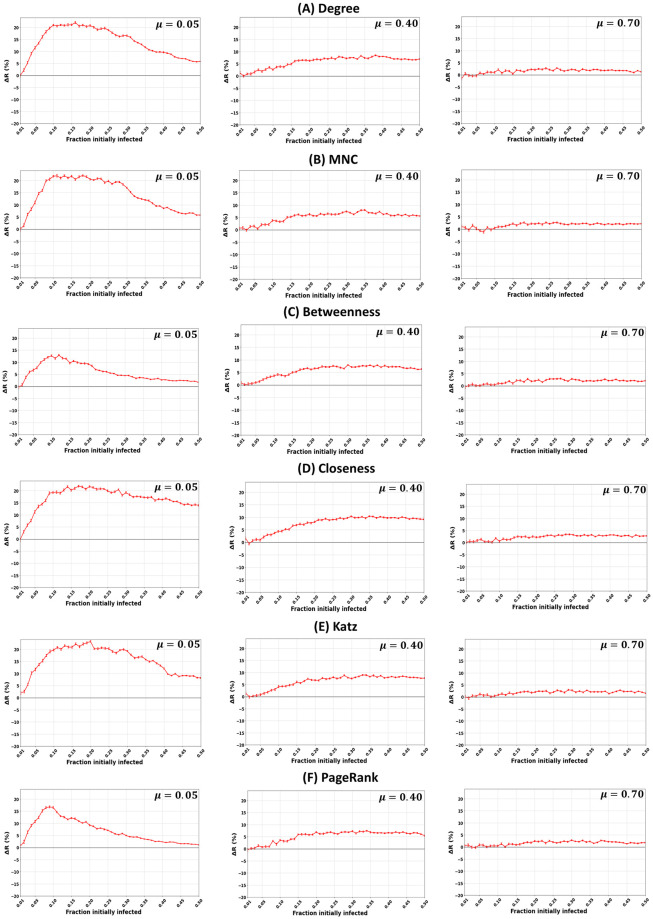
Impact of the community structure strength (*μ*) in synthetic networks. The figures represent the relative difference of the outbreak size (Δ*R*) as a function of the fraction of initially infected nodes. The red curve indicates the relative performance difference of the community-aware ranking strategy with the descending order ranking for the six centrality measures under test. The mixing parameter (*μ*) varies while the other parameters, including the community size distribution exponent (*θ* = 2.7) and the degree distribution exponent (*γ* = 2.7), are fixed.

Fig 2 shows the relative difference in the outbreak size (Δ*R*) as a function of the fraction of initially infected nodes of the six investigated centrality measures (Degree, Maximum Neighborhood Component, Betweenness, Closeness, Katz, PageRank) with a strong (*μ* = 0.05), medium (*μ* = 0.40), and weak (*μ* = 0.70) community structure strengths. The remaining parameters, including the community size (*θ*) and degree distribution (*γ*) exponents, are fixed at 2.7. The outbreak size (Δ*R*), represented by the red curve, is the difference between the number of nodes recovered after an initial set of nodes ranked based on the community-aware ranking scheme is infected and another initial set of nodes infected ranked based on the classical descending order ranking scheme. Thus, it represents a measure of performance of the community-aware ranking scheme. Positive values indicate that the proposed ranking scheme performs better (see S1 Text for details). The curves with all the values of *μ* are shown in S1 Fig.

In networks with a strong community structure (*μ* = 0.05), the community-aware ranking scheme always outperforms the classical descending order ranking scheme for all the centrality measures under study. The gain reaches 24% for Katz centrality at a fraction of initially infected nodes (*f*_*o*_) of 0.20, followed by 22% for Degree, MNC, and Closeness centrality. The performance of these measures is consistent from a fraction of initially infected nodes of 0.10 till 0.25, then they decline. Closeness centrality slightly declines, showing a Δ*R* of 14% at *f*_*o*_ = 0.50, followed by Katz centrality with 8%, then Degree and MNC obtaining a Δ*R* of 6%. Betweenness and PageRank are the less performing measures under the community-aware ranking scheme. The maximum gain for Betweenness is 12.5% at *f*_*o*_ = 0.12 and for PageRank is 16.5% at *f*_*o*_ = 0.9. After a peak, performance declines reaching a gain of 2% at *f*_*o*_ = 0.50.

In networks with a medium community structure (*μ* = 0.40), the community-aware ranking scheme of all the centrality measures still performs better than the classical descending order ranking scheme. When the fraction of initially infected nodes *f*_*o*_ is low (i.e., between 0.01 and 0.05), the gain for all the centrality measures is low, reaching a maximum of 1%. As the fraction of initially infected nodes increases, the performance of the community-aware ranking scheme also increases until it reaches a plateau or barely changes. For example, the relative difference in the outbreak size of Degree centrality increases from *f*_*o*_ equaling 0.10 to 0.25, going till Δ*R* = 6.5%, and then it hardly changes. MNC, Betweenness, Closeness, Katz, and PageRank show similar behavior with Δ*R* reaching a maximum between 5% and 10%.

In networks with a weak community structure (*μ* = 0.70), when the fraction of initially infected nodes is between 0.01 and 0.10, the relative difference in the outbreak size (Δ*R*) alternates between -1% and +1%. After that, it increases to a maximum of Δ*R* = 3% and shows a plateau. One can expect these results. Indeed, the frontier between weak community structure and no community structure is thin.

**Fig 3:**
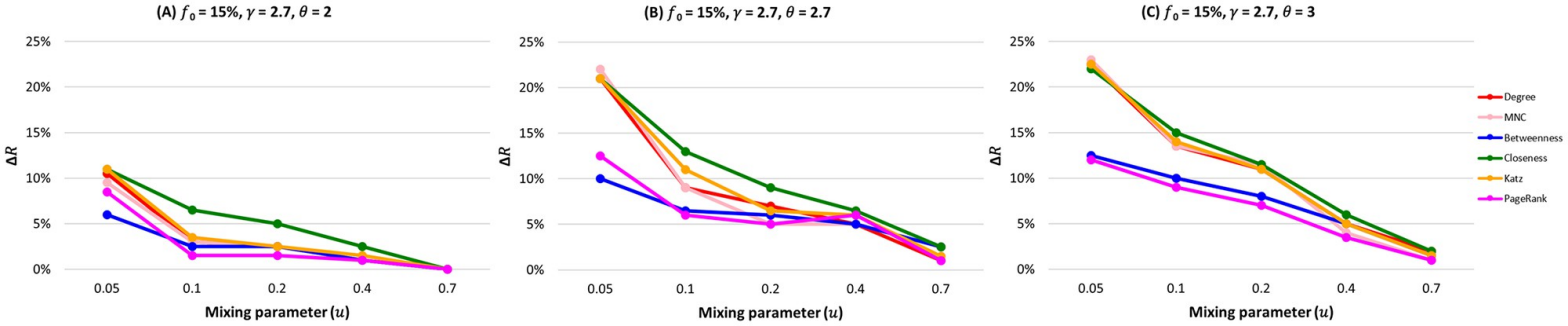
The relative difference of the outbreak size (Δ*R*) as a function of the mixing parameter (*μ*) when fraction of initially infected nodes (*f*_*o*_) equals 0.15. The color of the curve represents the centrality measures under study. (A) Synthetic networks with degree distribution *γ* = 2.7 and community size distribution *θ* = 2. (B) Synthetic networks with degree distribution *γ* = 2.7 and community size distribution *θ* = 2.7. (C) Synthetic networks with degree distribution *γ* = 2.7 and community size distribution *θ* = 3.

We fix the fraction of initially infected nodes at 0.15 for all the centrality measures in Fig 2 and plot the relative difference in the outbreak size (Δ*R*) as a function of the mixing parameter (*μ*) as shown in Fig 3B. As the community structure gets weaker (i.e., from *μ* = 0.05 to *μ* = 0.7), the performance of the community-aware ranking scheme starts declining. Moreover, one can differentiate between the centrality measures’ effectiveness. Closeness is the best-performing centrality measure, followed by Katz, Degree, and MNC. In contrast, Betweenness and PageRank perform poorly. However, all the measurements show a higher relative difference in the epidemic outbreak size than the classical descending order ranking scheme.

These results show that the community-aware ranking scheme is more effective in networks with a strong community structure. Indeed, in a strong community structure, communities are so well-separated that one can consider them independent subnetworks with their topological characteristics. In turn, targeting the most influential nodes in each community leads to a higher spreading, ensuring that the diffusion reaches all communities. As the community structure gets weaker, the performance of the community-aware ranking scheme decreases. Since the community structure is not well defined, the network is barely distinguishable from the network with no community structure. However, even in the worst-case scenario, the community-aware ranking scheme still is more effective than the classical descending order ranking scheme.

### Influence of the community size distribution

This investigation aims to analyze the impact of the community size distribution on the community-aware ranking scheme. One can tune the power-law community size distribution exponent (*θ*) in the networks generated by the LFR. In this study, we evaluate two values representing extreme cases. In the first case with *θ* = 2, few small communities coexist with large communities with a large variance in community sizes. In the second case, with *θ* = 3, more communities of equivalent sizes coexist, and the variance in the community sizes is minor. There are more communities in the second case than in the first case. Table 3 in S1 Text reports the number of communities of each generated network, along with the minimum and maximum size of the communities. The histograms of the distributions are given in S2 Fig. Note that we also perform tests with *θ* = 2.7. However, there were no significant differences compared to *θ* = 3.

**Fig 4:**
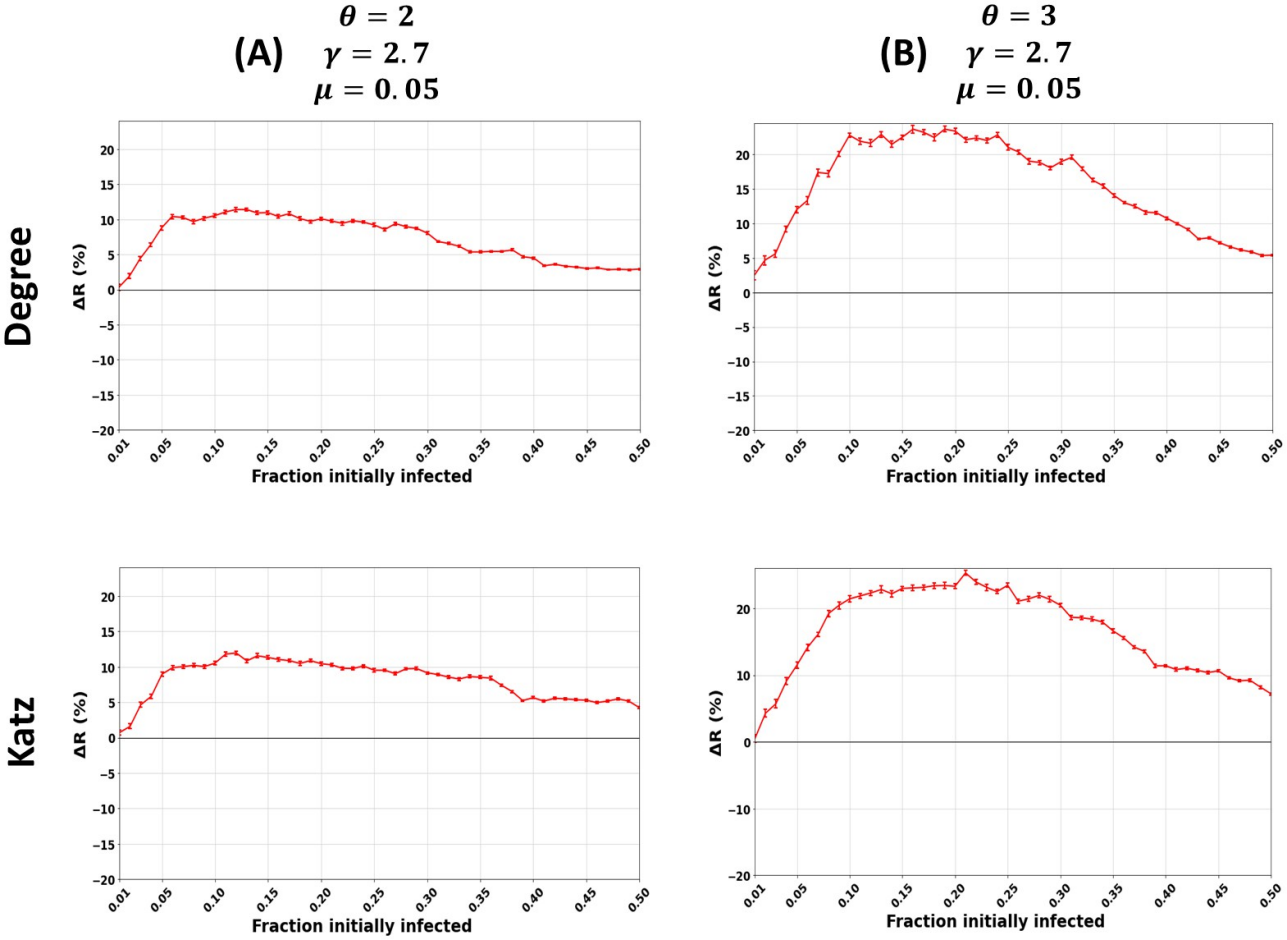
Impact of the community size distribution exponent (*θ*) in synthetic networks. The figures represent the relative difference of the outbreak size (Δ*R*) as a function of the fraction of initially infected nodes. The red curve indicates the relative performance difference of the community-aware ranks of the Degree and Katz centrality measures compared to the descending order ranks. The community size distribution exponent (*θ*) varies while the other parameters, including the mixing parameter (*μ* = 0.05) and the degree distribution exponent (*γ* = 2.7), are fixed.

Fig 4 shows the relative difference in the outbreak size (Δ*R*) as a function of the fraction of initially infected nodes for the Degree and Katz centrality. The community size distribution exponent *θ* equals 2 (panel A) and 3 (panel B). The other parameters are fixed, including the mixing parameter (*μ* = 0.05) and the degree distribution exponent (*γ* = 2.7). For the sake of brevity, since the remaining results of the centrality measures show similar behavior, they are given in S3 and S4 Figs with *θ* = 2 and *θ* = 3, respectively.

When *θ* = 2 (Fig 4A), the networks contain a few small communities coexisting with much larger ones. The relative difference of the outbreak size (Δ*R*) increases as the fraction of initially infected nodes increases reaching a maximum of 11% for Degree centrality and 12% for Katz centrality for the community-aware ranking scheme. Then, Δ*R* barely varies when the fraction of initially infected nodes *f*_*o*_ ranges from 0.10 and 0.25. After that, it starts to gradually decrease, reaching 3.5% and 4% for both centralities, respectively, when *f*_*o*_ = 0.50.

When *θ* equals 3 (Fig 4B), there are many small communities of comparable sizes and a few large ones. The performance of the community-aware ranking scheme for Degree centrality increases, reaching a maximum of 24% gain in terms of Δ*R*. Then it gradually decreases until it reaches 5.1% gain when the fraction of initially infected nodes is 0.50. Katz centrality exhibits similar behavior. The relative outbreak size increases as the fraction of initially infected nodes increases, reaching a maximum gain of 24%. It decreases until it reaches a gain of 8% when the fraction of initially infected nodes equals 0.50.

Fig 3 reports the relative outbreak size (Δ*R*) for all the centrality measures in synthetic networks with a community size distribution exponent (*θ*) spanning from 2 (Panel A) to 3 (Panel C). The fraction of initially infected nodes (*f*_*o*_) is fixed at 15%. When networks have a large difference in the sizes of the communities leading to fewer communities (*θ* = 2), the gain in Δ*R* of the community-aware ranking scheme ranges from 11% as a maximum at *μ* = 0.05. It decreases, reaching 0% when *μ* = 0.70. On the contrary, when the networks have many small communities with fewer larger ones leading to many communities, Δ*R* for Degree, MNC, Closeness, and Katz reach a gain of 23% and a gain of 13% and 12.7% for Betweenness and PageRank, respectively. As the community structure gets weaker, Δ*R* decreases to a minimum of 1% for PageRank centrality and a maximum of 2.3% for Closeness centrality.

Results indicate that when the network contains a few large communities, the community-aware ranking scheme is not as effective as in networks with many communities of smaller sizes. It is reasonable since when huge communities coexist with a few small communities, the large communities will make up most of the network. When one picks the top influential nodes from each community in the first iteration, the nodes picked in the second iteration inside the large communities are likely to be next to each other. Indeed, when there are substantial communities, there are few communities overall. Thus, infecting the most influential nodes in the same neighborhood is not as effective as covering many communities of comparable sizes with the community-aware ranking scheme.

### Influence of the degree distribution

In this experiment, we investigate the effect of the degree distribution on the performance of the community-aware ranking scheme. The degree distribution exponent (*γ*) is tunable in the LFR model. Studies have shown that real-world networks are scale-free, with a degree distribution exponent in the range of 2 and 3 [35, 36]. Consequently, we test these two values. Note that we also set *γ* = 2.7, but there are no significant differences compared to *γ* = 3. When *γ* equals 2, the network’s structure resembles a hub-and-spoke network [37]. When *γ* equals 3, the network’s structure is more similar to a random network where more nodes would have comparable frequency of neighbors. Since the LFR model generates also networks with a community structure, the nodes inside the communities have comparable sizes while assuring the community structure is maintained [32].

**Fig 5:**
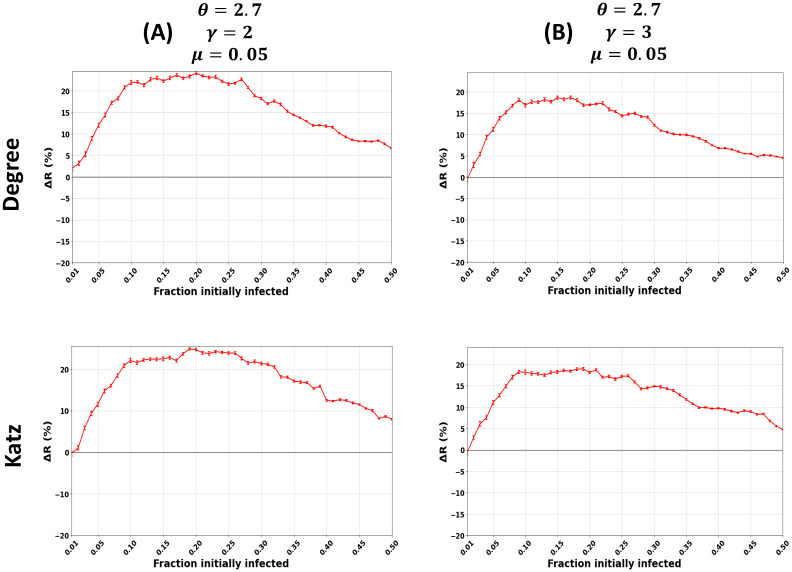
Impact of the degree distribution exponent (*γ*) in synthetic networks. The figures represent the relative difference of the outbreak size (Δ*R*) as a function of the fraction of initially infected nodes. The red curve indicates the relative performance difference of the community-aware ranks of the Degree and Katz centrality measures compared to the descending order ranks. The degree distribution exponent (*γ*) varies while the other parameters, including the mixing parameter (*μ* = 0.05) and the community size distribution exponent (*θ* = 2.7), are fixed.

Fig 5 shows the relative difference in the outbreak size (Δ*R*) as a function of the fraction of initially infected nodes for the Degree and Katz centrality. The degree distribution exponent *γ* equals 2 (panel A) and 3 (panel B). We fix all the other parameters, including the mixing parameter (*μ* = 0.05) and the community size distribution exponent (*θ* = 2.7). For the sake of brevity, since the remaining results of the centrality measures exhibit similar behavior, they are given in S5 and S6 Figs with *γ* = 2 and *γ* = 3, respectively.

When *γ* = 2 (Fig 5A), generating networks with a hub-and-spoke structure, the relative difference of the outbreak size (Δ*R*) of both Degree centrality and Katz centrality under the community-aware ranking scheme escalates quickly from fraction of initially infected nodes (*f*_*o*_) amounting to 0.01 till 0.10, reaching a maximum of 24%. Δ*R* stays in this range between 20% and 24% from *f*_*o*_ = 0.11 till *f*_*o*_ = 0.27 for Degree and *f*_*o*_ = 0.30 for Katz. After which Δ*R* starts to decrease reaching 6.5% for Degree and 8.5% for Katz at *f*_*o*_ = 0.50.

When *γ* = 3, the degree distribution of the communities of the generated networks is more random than average. One can observe that both Degree centrality and Katz centrality perform similarly according to the relative difference of the outbreak size (Δ*R*). Compared to the descending order ranking scheme, the gain of the community-aware ranking scheme reaches 19% and barely changes till *f*_*o*_ = 0.25. Then it starts to gradually decrease reaching Δ*R* = 5% at *f*_*o*_ = 0.50.

**Fig 6:**
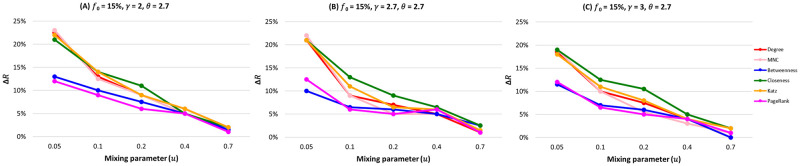
The relative difference of the outbreak size (Δ*R*) as a function of the mixing parameter (*μ*) when fraction of initially infected nodes (*f*_*o*_) equals 0.15. The color of the curve represents the centrality measures under study. (A) Synthetic networks with degree distribution *γ* = 2 and community size distribution *θ* = 2.7. (B) Synthetic networks with degree distribution *γ* = 2.7 and community size distribution *θ* = 2.7. (C) Synthetic networks with degree distribution *γ* = 3 and community size distribution *θ* = 2.7.

Fig 6 reports the differences in the relative outbreak size (Δ*R*) for all the centrality measures in networks with a degree distribution exponent (*γ*) spanning from 2 (Panel A) to 3 (Panel C). The fraction of initially infected nodes (*f*_*o*_) equals 15% When networks are similar to a hub-and-spoke structure (Fig 6A) with a strong community structure (*μ* = 0.05), centrality measures under the community-aware ranking scheme always show a higher relative outbreak size difference (Δ*R*). However, one can consider two categories. The first, including Degree, MNC, Closeness, and Katz, exhibit gains ranging from 21% and 23.5%. The second involving Betweenness and PageRank obtains a gain of around 13%. As we shift to a more random-like structure (*γ* = 3) in Fig 6C, the categorization of the centrality measures observed at *γ* = 2 remains the same. However, for the first category, the gain decreases between 17% and 18%. The second category exhibits a gain of around 11%. As the community structure weakens, the difference in the outbreak size becomes less pronounced. Nevertheless, the community-aware ranking scheme always performs better than the descending order ranking scheme.

Even though the differences are not as pronounced compared to the variation in the community size distribution, results show that when the communities of the generated networks are more random-like, the performance of the community-aware ranking scheme slightly decreases. Since more nodes have a comparable number of connections internally in a random-like structure, they may have similar centrality values. In turn, the community-aware ranking scheme may be prone to selecting nodes of the same influence inside their communities, saturating the diffusion spread. On the contrary, in a network with well-separated communities such as the hub-and-spoke structure, a community-aware ranking scheme can distinctively pick influential nodes in their communities that are naturally not close to each other due to the hub-and-spoke structure. It results in a higher diffusion to more isolated areas that the descending order ranking scheme cannot reach.

## Real-world networks

We also investigate the community-aware ranking scheme on 33 real-world networks covering a wide range of domains (i.e., infrastructural, biological, social, collaboration, and ecological). Since their community structure is unknown, we uncover it using two community detection algorithms: Infomap [38] and Louvain [39]. It allows us to check the impact of the community structure variations on the consistency of the community-aware ranking scheme. Table 4 in S1 Text reports the values of the topological characteristics of the real-world networks.

### Spreading power of the proposed method

**Fig 7:**
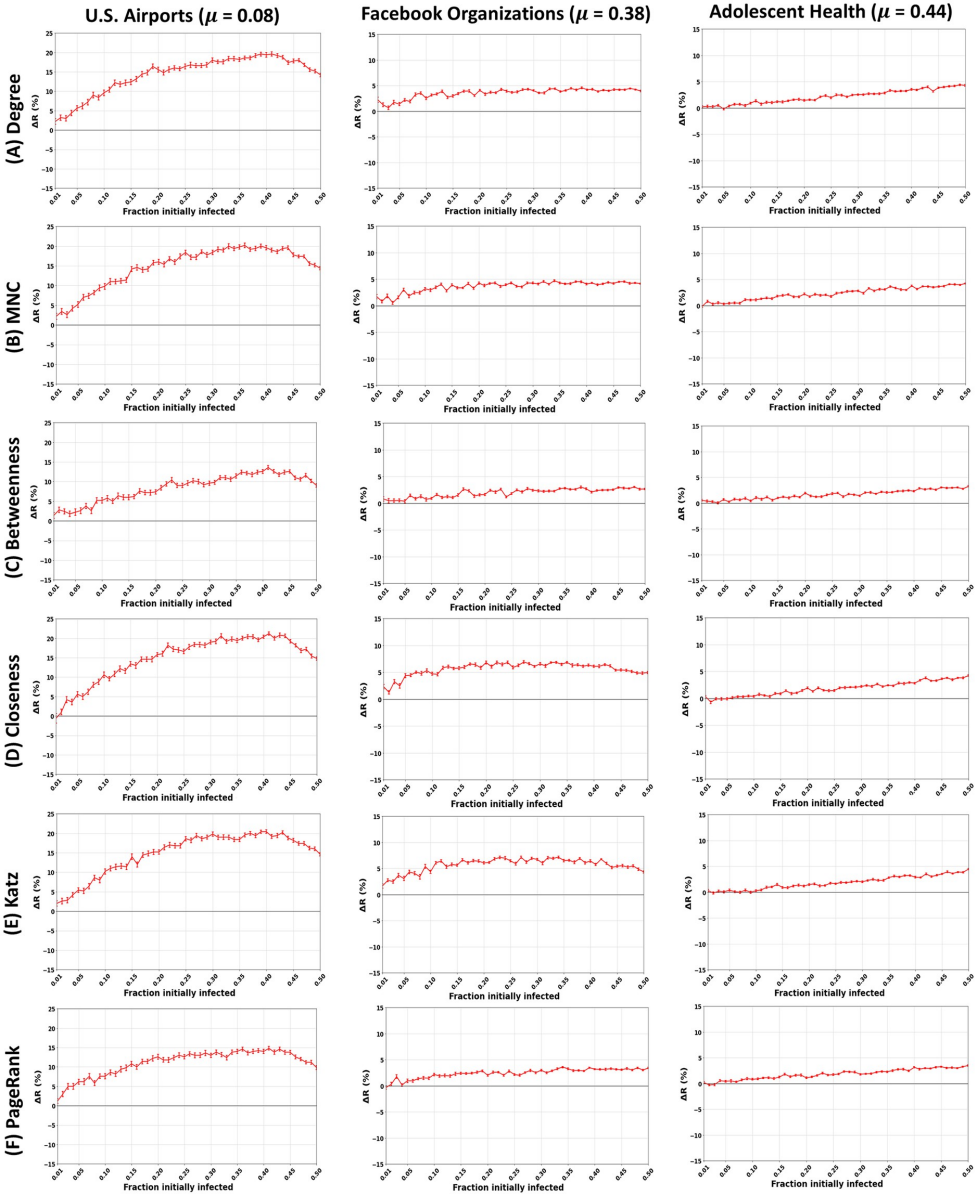
Impact of the community structure strength (*μ*) in real-world networks. The figures represent the relative difference of the outbreak size (Δ*R*) as a function of the fraction of initially infected nodes. The red curve indicates the relative performance difference of the community-aware ranking strategy with the descending order ranking for the six centrality measures under test. A strong, medium, and weak mixing parameter (*μ*) is derived based on the communities in real-world networks (U.S. Airports, Facebook Organizations, and Adolescent Health) identified by the Infomap community detection algorithm.

Since the community structure strength is a significant feature influencing the performance of the proposed community-aware ranking strategy, we classify the networks into three categories. The categories cover networks with strong (*μ* ≤ 0.20), medium (0.20 < *μ* < 0.40), and weak (*μ* ≥ 0.40) community structures. We consider communities uncovered by Infomap as our reference case. For the sake of brevity, we report one network of each category for all the centrality measures under study in Fig 7. The remaining networks are provided in S7–S13 Figs.

**Fig 8:**
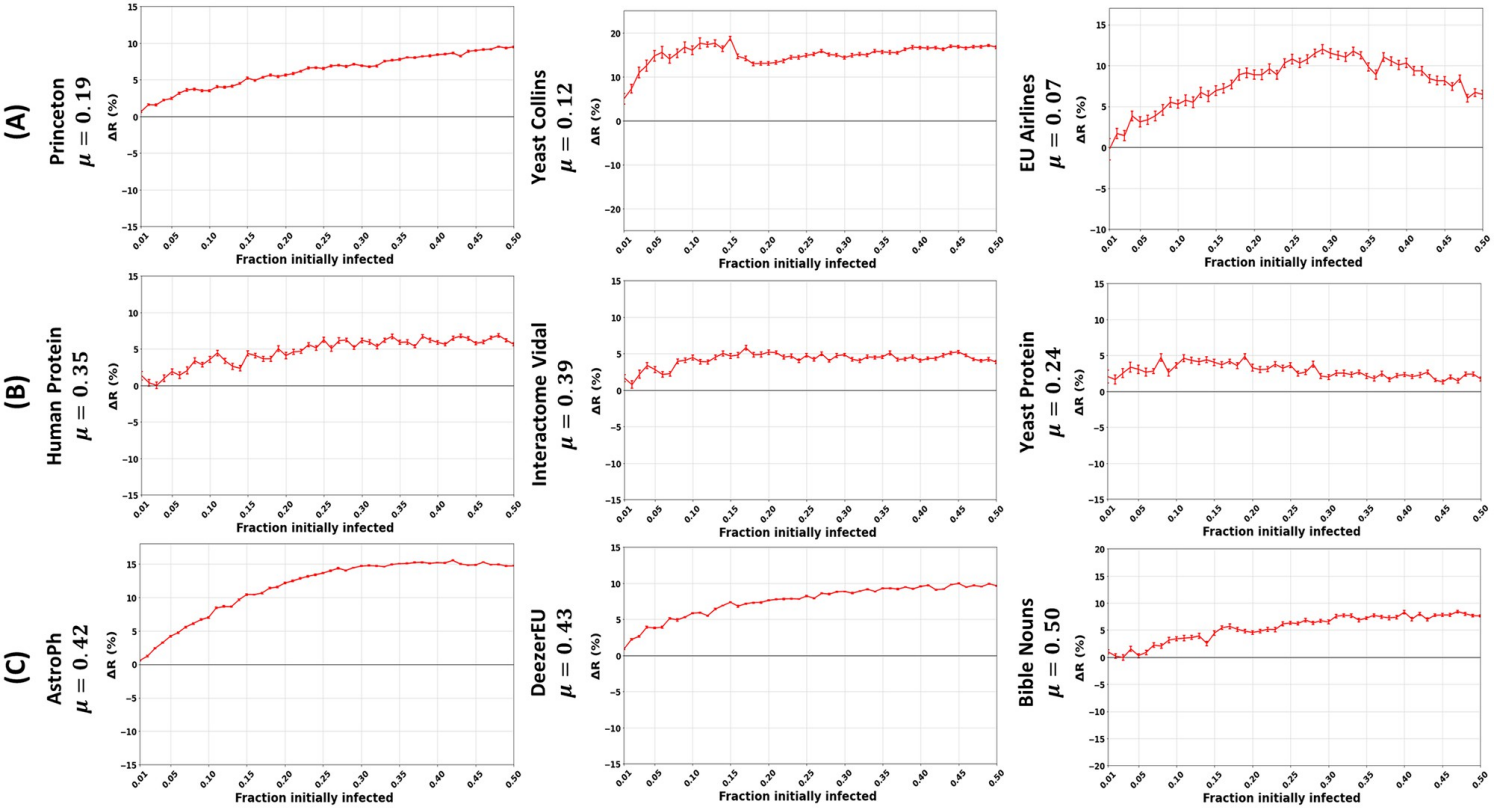
Trends in the performance of the community-aware ranking scheme in real-world networks. The figures represent the relative difference of the outbreak size (Δ*R*) as a function of the fraction of initially infected nodes. The red curve indicates the relative performance difference of the community-aware ranks of the Degree centrality compared to the descending order ranks. Communities are identified using Infomap. (A) Networks with a strong community structure strength. (B) Networks with a medium community structure strength. (C) Networks with a weak community structure strength.

The community-aware ranking scheme outperforms the classical descending order ranking scheme in networks with a strong community structure (*μ*≤ 0.20). The distinction lies in the gain in the relative difference of the outbreak size (Δ*R*). As depicted by U.S. Airports network (with *μ* = 0.08) in Fig 7, one can note the outperformance of Closeness centrality, with a difference in the outbreak size (Δ*R*) reaching a maximum of 21% when the fraction of initially infected nodes (*f*_*o*_) amounts to 0.41, followed by Degree centrality with Δ*R* = 20% at *f*_*o*_ = 0.41 and MNC and Katz with Δ*R* = 20% at *f*_*o*_ = 0.40. Then comes PageRank with Δ*R* = 15% at *f*_*o*_ = 0.41 followed by Betweenness with Δ*R* = 14% at *f*_*o*_ = 0.41. In general, in all the networks, Closeness, Degree, MNC, and Katz show higher Δ*R* compared to Betweenness and PageRank. One can also note three typical behaviors for the performance of the community-aware ranking scheme in networks with a strong community structure. These behaviors are common to all the centrality measures within a given network. For brevity, we report the results of Degree centrality only. The first typical behavior is that Δ*R* increases as *f*_*o*_ increases. The remaining figures are provided in the supplementary material. It is illustrated by the Princeton network in Fig 8A on the left. Ego Facebook, Facebook Friends, and Facebook Politician Pages share similar behavior. The second typical behavior is that Δ*R* increases until it reaches a plateau or barely deviates as *f*_*o*_ increases. It is shown in the middle of Fig 8A for the Yeast Collins network. London Transport, Malaria Genes, NetSci, Board of Directors, and DNC Emails show similar behavior. Finally, in the third case, Δ*R* increases until it reach a specific value of *f*_*o*_, then decreases gradually. It is demonstrated by the EU Airlines network in Fig 8A on the right. U.S. Airports, Madrid Train Bombings, Reptiles, 911 All Words, Marvel Partnerships, U.S. Power Grid, PGP, EuroRoad, and Internet Topology Cogentco share similar behavior.

The community-aware ranking scheme still outperforms the classical descending order ranking scheme in networks with a medium community structure (0.20 <*μ*< 0.40). However, the gain is less pronounced compared to networks with a strong community structure. It is depicted by the Facebook Organizations network in Fig 7. One can see that Closeness and Katz centrality measures achieve the highest gain in Δ*R* amounting to 6% and 7% from a fraction of initially infected nodes amounting to 0.15 till 0.45. For Degree and MNC, the maximum gain is 4% and 4.5%, respectively. Then, Betweenness and PageRank show a gain of Δ*R* = 2.5% and Δ*R* = 3.5, respectively. One can also note that within this category, we observe three behaviors for the performance of the community-aware ranking scheme. These behaviors are similar to those in networks with a strong community structure but at a smaller magnitude. We have an increasing Δ*R* as *f*_*o*_ increases, depicted by the Human Protein network on the left of Fig 8B. Hamsterster and Blumenau Drug share similar behavior. In the second category, we have an increasing Δ*R* until it reaches a plateau or barely deviates as *f*_*o*_ increases. It is depicted by the Interactome Vidal network in the middle of Fig 8B. Facebook Organizations and Caltech share similar behavior. Finally, the third category shows a slight and gradual decrease directly from the start as *f*_*o*_ increases. It is illustrated by the Yeast Protein network in Fig 8B on the right. Retweets Copenhagen shows similar behavior.

The community-aware ranking scheme outperforms the classical descending order ranking scheme in networks with a weak community structure (*μ* ≥ 0.40). However, in some networks, the gain in Δ*R* can even be higher than in networks with a strong or medium community structure. Indeed, the maximum improvement in Δ*R* can reach up to 15% in the AstroPh network (see Fig 8C) with Degree, MNC, Closeness, and Katz centrality measures and up to 11% and 10% for PageRank and Betweenness centrality measures respectively. At the same time, it can be as low as 5% in Adolescent Health given in Fig 7 for Degree, MNC, Closeness, and Katz and as low as 4% for Betweenness and 3% for PageRank. That being said, in networks with a weak community structure there is one trend despite the difference in magnitude. Indeed, as seen from Fig 8C, all networks (AstroPh, DeezerEU, and Bible Nouns) have an increasing Δ*R* as *f*_*o*_ increases. The only difference is in the magnitude of Δ*R* from one network to another.

In summary, the community-aware ranking scheme outperforms the descending order ranking scheme in all real-world networks under study. The gain of the proposed ranking scheme is affected by the community structure strength, as observed in artificial networks with controlled community structure strength. The stronger the community structure, the higher the performance of the community-aware ranking scheme. Nevertheless, it is worth noting that the community-aware ranking also shows high performance in some real-world networks with a weak community structure.

**Fig 9:**
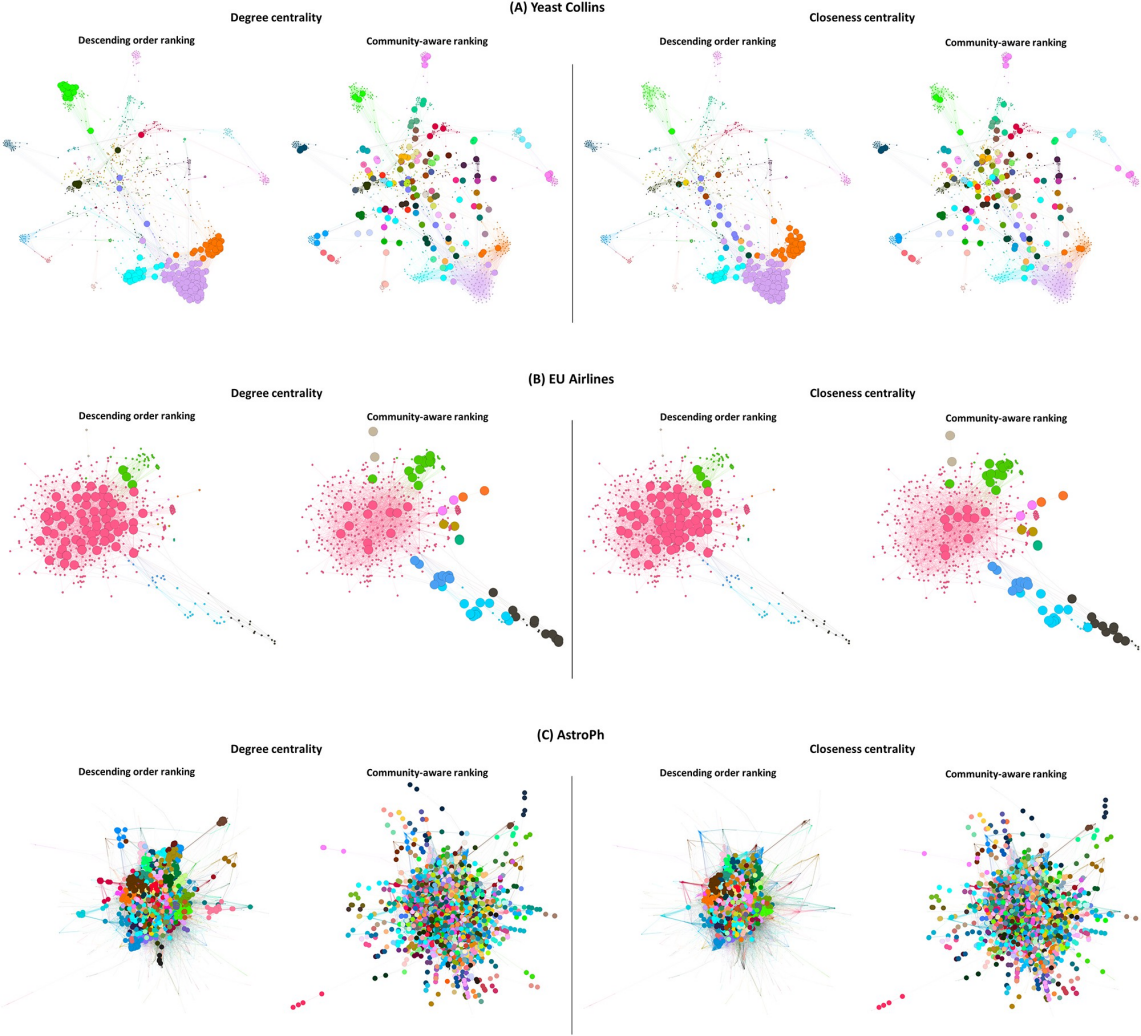
Top nodes selected based on the Degree and Closeness centrality measures according to the descending order and community-aware ranking schemes. Communities of the Yeast Collins (A), EU Airlines (B), and AstroPh (C) networks are identified by Infomap. The top selected nodes (depicted in bigger sizes) amount to 15% of the network’s size.

The community-aware ranking scheme has a high performance in networks with strong community structure strength because it does not select nodes in one dense region when there are many well-separated dense areas. We visualize two networks with a strong community structure strength but with different topological structures, namely Yeast Collins and EU Airlines in Fig 9A and 9B, respectively. In these two networks, we pick and increase the size of the top 15% of nodes selected by the descending order ranking scheme and the community-aware ranking scheme. For brevity, we only show Degree centrality and Closeness centrality. Concerning the Yeast Collins network, for both Degree and Closeness centrality, one can directly point out how the descending order scheme selects most of the top nodes in large network communities mainly located at the bottom of the network. On the contrary, the community-aware ranking scheme selects nodes in every community, spreading across all the network regions. A similar interpretation goes for the EU Airlines network, another network with a strong community structure. Indeed, the descending order ranking scheme of Degree and Closeness centrality measures targets only the dark pink and green communities. In contrast, the community-aware ranking scheme does not miss a single community. It is the reason why the community-aware ranking strategy allows a higher diffusion.

We also investigate the case of networks with a weak community structure where the proposed community-aware ranking scheme performs well. Fig 9C visualizes the AstroPh network, a network with a weak community structure and high performance of the community-aware ranking scheme. Despite having loosely defined communities with a vast number of inter-community connections, the community-aware ranking scheme targets nodes at the core and in the periphery of the AstroPh network, either with Degree or Closeness centrality measures. While with the descending order ranking scheme, using Degree or Closeness centrality measures, nodes picked are mainly in the network’s core. Consequently, the community-aware ranking scheme can ignite a higher diffusion as it reaches regions that the descending order ranking scheme never targets.

It is worth mentioning that networks characterized by a weak community structure may exhibit different topologies [40, 41]. If it is core-periphery-like, such as in the AstroPh network, the community-aware ranking scheme covers all the regions in the network. If the network is very dense with no particular local structure, the community-aware ranking scheme might select nodes in the vicinity of each other. We suggest to use a measure that combines local and global influence of the nodes for better targeting influential nodes [42, 43]. Note that the distance between the nodes should also be addressed. Alternatively, one can also incorporate a minimum distance constraint between nodes in a community so that targeted nodes are scattered. There is room for improvement in networks with a weak community structure.

### Influence of the community detection algorithm

In this experiment, we use the Louvain [39] community detection algorithm to extract the communities in real-world networks. Then, we perform the same comparative evaluation process using SIR simulations between the classical and the proposed ranking strategies based on the communities identified by Louvain instead of Infomap [38]. The aim is to investigate the impact of the variations in the community structure induced by the community detection algorithms on the performance of the ranking schemes.

**Fig 10:**
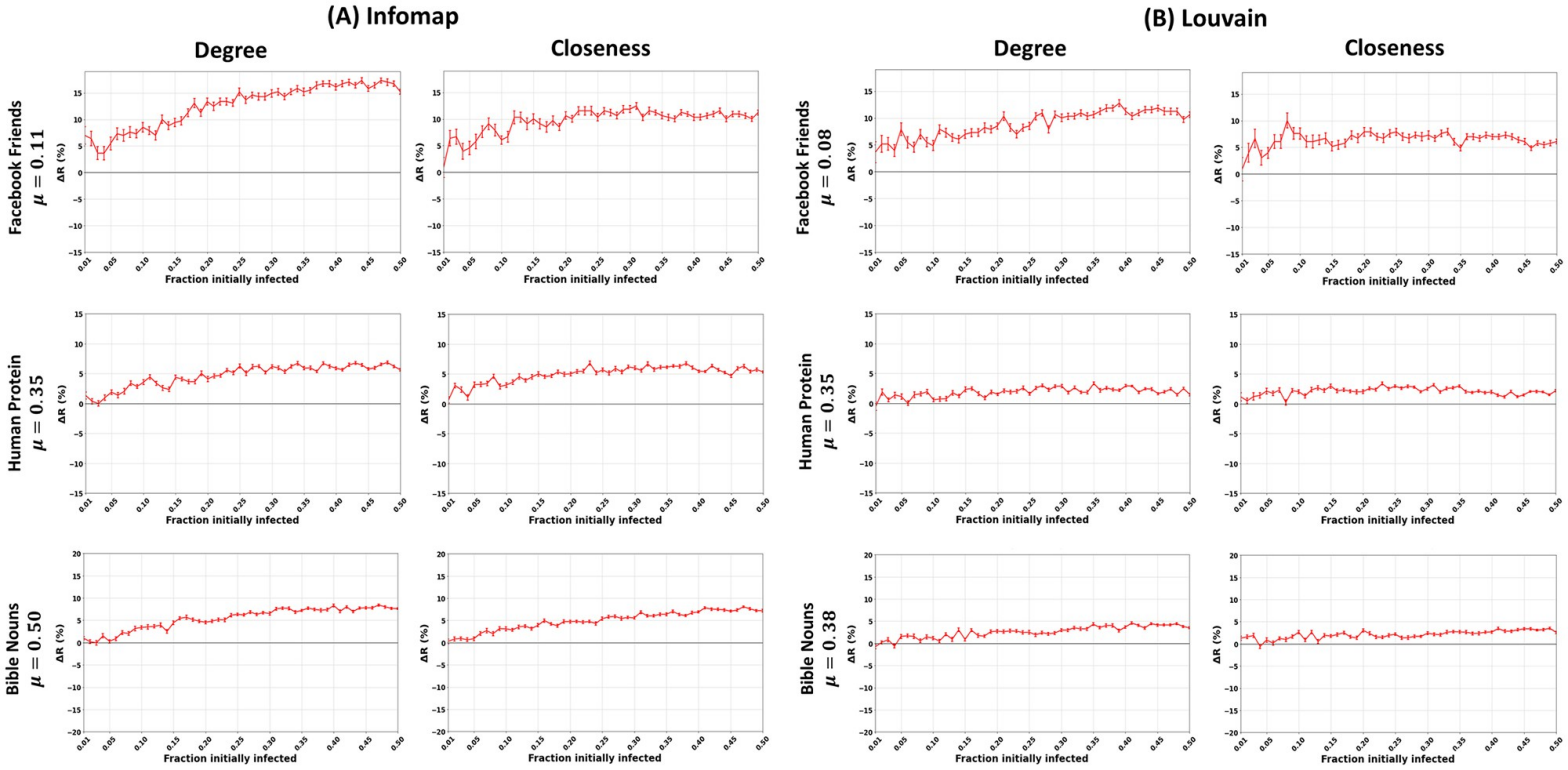
Impact of the community detection algorithm in real-world networks with strong, medium, and weak community structure strengths. The figures represent the relative difference of the outbreak size (Δ*R*) as a function of the fraction of initially infected nodes. The red curve indicates the relative performance difference of the community-aware ranks of the Degree and Closeness centrality measures compared to the descending order ranks. (A) Communities identified using Infomap. (B) Communities identified using Louvain.

Fig 10 illustrates the relative difference in the outbreak size (Δ*R*) of the community-aware ranking scheme for Degree and Closeness centrality measures. We comment on the results for three typical networks (Facebook Friends, Human Protein, and Bible Nouns). Facebook Friends and Human Protein belong respectively to the strong and medium community structure categories using Infomap or Louvain. Bible Nouns network is in the medium community structure category based on Louvain and the weak community structure using Infomap. We provide complementary results for all the networks and all the centrality measures in S14–S20 Figs.

First, these results demonstrate that the community-aware ranking scheme is robust to the community structure variation induced by the community detection algorithm. Indeed, Δ*R* is positive whether Infomap or Louvain uncovers communities. This result is independent of the community structure strength. For example, Facebook Friends is a network with a strong community structure. For a fraction of initially infected nodes equal to 0.25, the gain of Δ*R* equals 15% for Degree centrality and 11% for Closeness centrality. It compares to an 8% increase for Degree centrality and 7% for Closeness centrality using Louvain with the same fraction of initially infected nodes. Consider the Human Protein network with a medium community structure strength. With a fraction of initially infected nodes equal to 0.25, the Δ*R* gain is 6% for Degree and Closeness centrality measures. In the same situation, using Louvain, the growth is lower. Indeed, Δ*R* equals 2% for Degree centrality and 3% for Closeness centrality. Finally, the community-aware ranking scheme still outperforms the classical descending order ranking scheme in the Bible Nouns network. For a fraction of initially infected nodes of 0.25, Δ*R* equals 6% for Degree centrality and 5.5% for Closeness centrality. Using Louvain with the same fraction of initially infected nodes reduces the gain in Δ*R* to 2.5% for Degree centrality and 2% for Closeness centrality.

Second, one can note that with Infomap, the gain in Δ*R* is relatively higher than Louvain. For instance, in Facebook Friends with communities identified using Infomap, the maximum Δ*R* reaches 17.5% at a fraction of initially infected nodes of 0.44 using Degree centrality and 13% using Closeness at a fraction of initially infected nodes of 0.31. In contrast, with the Louvain community detection algorithm, the maximum Δ*R* reaches 12.5% at a fraction of initially infected nodes of 0.39 using Degree centrality and 10% using Closeness at a fraction of initially infected nodes of 0.08. Thus, the difference in gain of Δ*R* is +5% for Degree centrality and +3% for Closeness centrality using Infomap. One observes similar results for Human Protein and Bible Nouns. The maximum gain in Δ*R* in Human Protein amounts to 6% using both Degree and Closeness centrality measures. Meanwhile, Louvain’s maximum gain in Δ*R* amounts to 3% for the two centrality measures. In Bible Nouns, with Infomap, the maximum improvement in Δ*R* amounts to 8.5% using both Degree and Closeness centrality measures. In opposition, using Louvain, the maximum gain in Δ*R* is 4.8% for Degree centrality and 3.8% for Closeness centrality.

**Fig 11:**
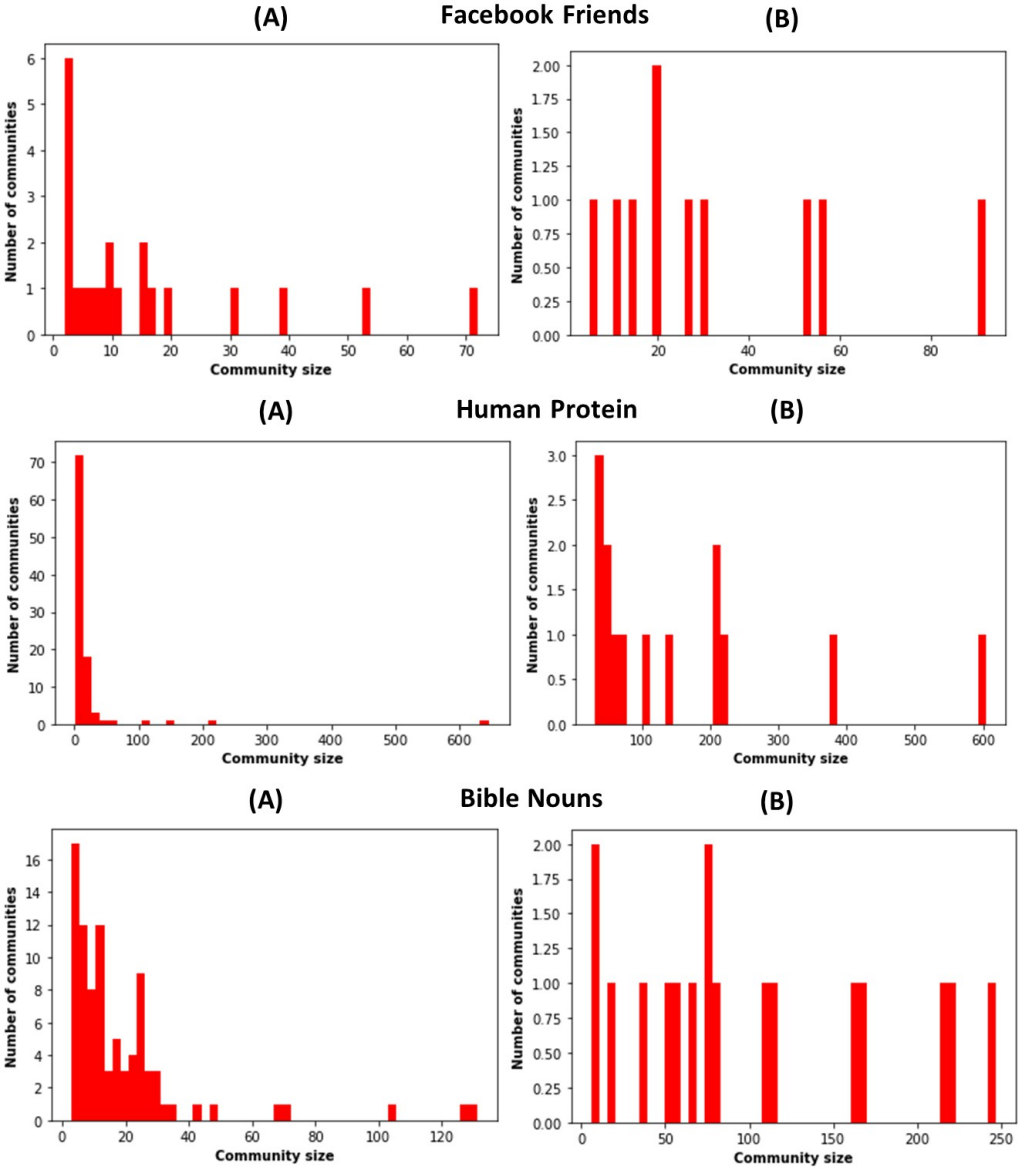
Histograms of the community size distribution. Communities are identified in Facebook Friends, Human Protein, and Bible Nouns by the Infomap (A) and Louvain (B) community detection algorithms.

To investigate why the performance of the community-aware ranking scheme decreases with Louvain compared to Infomap, we examine the community size distributions of the networks. Fig 10 gives the community size distributions associated with Infomap and Louvain of the networks provided in Fig 11. Indeed, they represent typical cases. The other distributions are given in S21–S23 Figs. Simultaneously, we compute the number of communities uncovered by each community detection algorithm and the minimum and maximum size of the communities for all the networks. Tables 5 and 6 in S1 Text report the results using Infomap and Louvain, respectively.

The histograms of the community size distributions in Fig 11 show that they are generally more skewed to the right using Infomap. A high number of small communities coexists with very few large communities. In contrast, this distribution is more uniform for the Louvain community structure. We observe many communities with medium and large sizes. Moreover, Infomap uncovers a higher number of communities. For example, it reveals 21 communities in Facebook Friends, 99 in Human Protein, and 88 in Bible Nouns. It compares with 10 communities in Facebook Friends, 14 in Human Protein, and 17 in Bible Nouns identified by Louvain.

The question is, how do these two outcomes affect the performance of the proposed community-aware ranking scheme compared to the classical descending order ranking scheme. On the one hand, Louvain generally uncovers fewer communities, with many medium-sized and large communities making up most of the network. On the other hand, Infomap discovers many more communities with a high number of small communities coexisting with fewer larger ones. The proposed community-aware ranking strategy is more effective with Infomap. Naturally, with more communities, it selects at least one node in every community. The higher the number of communities, the higher its ability to choose distinct nodes. It is not necessarily true with fewer communities. Indeed, first, one selects a node in each community, then at the next iteration, nodes are targeted in the same communities. Thus, nodes inside the same communities are probably closer to each other than when there are many communities. Thus, the diffusion power weakens. Indeed, it does not reach distant regions in the network, and the diffusion stays confined in large communities. Note that these results complement those reported using the generated LFR networks with controlled community size distribution exponent regarding the number of communities. Indeed, when fewer communities exist, the community-aware ranking scheme is more susceptible to picking the next top node close to the former top-picked node inside these communities. Consequently, infecting the most influential nodes in fewer communities is not as efficient as having many communities spanning the whole network.

## Conclusion

This paper presents a community-aware ranking scheme that one can use with any centrality measure. The proposed method is simple yet effective at selecting nodes according to their relative influence in a modular network. Unlike the popular descending order ranking scheme, which ranks the most influential nodes from high to low centrality values, it ranks nodes in a sequential order linked to the community size. Consequently, it selects nodes across all regions of the networks. In contrast with the descending order ranking scheme that can select nodes in a few communities that ignore large parts of the network, this strategy targets nodes more uniformly distributed. As a result, the proposed strategy warrants that the diffusion process does not die out locally and reaches distant regions of the network.

Extensive experiments have been conducted on synthetic and real-world networks using the SIR epidemic spreading model. To better understand the interplay between the community structure and the performance of the proposed strategy, we performed a series of experiments in synthetic networks controlling the community structure strength, the exponents of the community size, and degree distributions. Results show that the community-aware ranking scheme is more effective in networks with strong community structure strength. As it weakens, the performance decreases gradually. Nevertheless, the community-aware ranking scheme always performs better than the descending order ranking scheme, even in networks with a weak community structure. The community size distribution also affects the performance of the community-aware ranking scheme. Results show the strategy performs better in networks of many small communities instead of a few large communities. Indeed, the higher the number of communities, the more likely the targeted nodes are scattered across the network regions, igniting a higher epidemic outbreak. The influence of the degree distribution exponent is less pronounced. However, one can notice that the community-aware ranking scheme performs better in hub-and-spoke-like networks than in random-like ones.

The community-aware ranking scheme also outperforms the classical ranking strategy in a set of real-world networks of various domains. The findings are consistent with the synthetic networks’ experiments. Indeed, the community-aware ranking scheme performs better in networks with a strong community structure strength. The gain gradually decreases with the community structure strength. Note that in some networks with a weak community structure strength, the community-aware ranking scheme still creates a higher outbreak than its alternative. Indeed, the community-aware ranking scheme ranks the top nodes inside each community. Even in networks with a weak community structure, it can rank nodes in faraway regions, causing a higher outbreak. We also investigate the influence of the community detection algorithm on the performance of the community-aware ranking scheme. The comparisons involve Infomap and Louvain. Since the community structure uncovered by Louvain results in fewer communities and subsequently larger ones compared to Infomap, the community-aware ranking scheme performs better with the Infomap community structure. This result is coherent with synthetic networks’ community size distribution variation. Whatever the community detection algorithm, the community-aware ranking scheme consistently outperforms the descending order ranking strategy.

The main lesson from this study is to highlight the necessity of incorporating the community structure information in centrality measurements to better rank influential nodes. This work departs from previous community-aware solutions that combine a node’s local influence and global influence. Here, we show that whatever the notion of influence, the ranking strategy is a critical factor in the diffusion process. Whatever the centrality measure, the proposed ranking scheme is decisive in targeting the most influential nodes scattered across the network. This strategy overcomes the drawback frequent in centrality measures using the popular descending order ranking scheme in which the most influential nodes happen to be in the vicinity of each other. The proposed ranking scheme is adequate for igniting higher diffusion for marketing and awareness campaigns or combating diseases and unwanted viruses since it pinpoints influential nodes while assuring that all regions in the network are covered.

In future work, we plan to develop a more sophisticated community-aware ranking scheme to overcome the decrease in performance when there are few and large communities. Another research direction consists of adapting the strategy to networks with an overlapping community structure. Furthermore, one can consider community-aware ranking schemes for multilayer and temporal networks.

## Supporting information

S1 Fig Impact of the community structure strength (*μ*) in synthetic networks. The figures represent the relative difference of the outbreak size (Δ*R*) as a function of the fraction of initially infected nodes. The red curve indicates the relative performance difference of the community-aware ranking strategy with the descending order ranking for the six centrality measures under test. The mixing parameter (*μ*) is varied while the other parameters, including the community size distribution exponent (*θ* = 2.7) and the degree distribution exponent (*γ* = 2.7), are fixed.
(PNG)

S2 Fig The histograms of the community size distribution for the synthetic networks. The synthetic networks are generated with *θ* = 2 and *θ* = 3 from strong to weak community structure strengths while keeping other parameters fixed including the degree distribution exponent (*γ* = 2.7).
(PNG)

S3 Fig Impact of the community size distribution exponent (*θ*) in synthetic networks. The figures represent the relative difference of the outbreak size (Δ*R*) as a function of the fraction of initially infected nodes. The red curve indicates the relative performance difference of the community-aware ranking strategy with the descending order ranking for the six centrality measures under test. The mixing parameter (*μ*) is varied while the other parameters, including the community size distribution exponent (*θ* = 2) and the degree distribution exponent (*γ* = 2.7), are fixed.
(PNG)

S4 Fig Impact of the community size distribution exponent (*θ*) in synthetic networks. The figures represent the relative difference of the outbreak size (Δ*R*) as a function of the fraction of initially infected nodes. The red curve indicates the relative performance difference of the community-aware ranking strategy with the descending order ranking for the six centrality measures under test. The mixing parameter (*μ*) is varied while the other parameters, including the community size distribution exponent (*θ* = 3) and the degree distribution exponent (*γ* = 2.7), are fixed.
(PNG)

S5 Fig Impact of the degree distribution exponent (*γ*) in synthetic networks. The figures represent the relative difference of the outbreak size (Δ*R*) as a function of the fraction of initially infected nodes. The red curve indicates the relative performance difference of the community-aware ranking strategy with the descending order ranking for the six centrality measures under test. The mixing parameter (*μ*) is varied while the other parameters, including the community size distribution exponent (*θ* = 2.7) and the degree distribution exponent (*γ* = 2), are fixed.
(PNG)

S6 Fig Impact of the degree distribution exponent (*γ*) in synthetic networks. The figures represent the relative difference of the outbreak size (Δ*R*) as a function of the fraction of initially infected nodes. The red curve indicates the relative performance difference of the community-aware ranking strategy with the descending order ranking for the six centrality measures under test. The mixing parameter (*μ*) is varied while the other parameters, including the community size distribution exponent (*θ* = 2.7) and the degree distribution exponent (*γ* = 3), are fixed.
(PNG)

S7 Fig Impact of the community structure strength (*μ*) in real-world networks. The figures represent the relative difference of the outbreak size (Δ*R*) as a function of the fraction of initially infected nodes. The red curve indicates the relative performance difference of the community-aware ranking strategy with the descending order ranking for the six centrality measures under test. The community structure is identified by the Infomap community detection algorithm.
(PNG)

S8 Fig Impact of the community structure strength (*μ*) in real-world networks. The figures represent the relative difference of the outbreak size (Δ*R*) as a function of the fraction of initially infected nodes. The red curve indicates the relative performance difference of the community-aware ranking strategy with the descending order ranking for the six centrality measures under test. The community structure is identified by the Infomap community detection algorithm.
(PNG)

S9 Fig Impact of the community structure strength (*μ*) in real-world networks. The figures represent the relative difference of the outbreak size (Δ*R*) as a function of the fraction of initially infected nodes. The red curve indicates the relative performance difference of the community-aware ranking strategy with the descending order ranking for the six centrality measures under test. The community structure is identified by the Infomap community detection algorithm.
(PNG)

S10 Fig Impact of the community structure strength (*μ*) in real-world networks. The figures represent the relative difference of the outbreak size (Δ*R*) as a function of the fraction of initially infected nodes. The red curve indicates the relative performance difference of the community-aware ranking strategy with the descending order ranking for the six centrality measures under test. The community structure is identified by the Infomap community detection algorithm.
(PNG)

S11 Fig Impact of the community structure strength (*μ*) in real-world networks. The figures represent the relative difference of the outbreak size (Δ*R*) as a function of the fraction of initially infected nodes. The red curve indicates the relative performance difference of the community-aware ranking strategy with the descending order ranking for the six centrality measures under test. The community structure is identified by the Infomap community detection algorithm.
(PNG)

S12 Fig Impact of the community structure strength (*μ*) in real-world networks. The figures represent the relative difference of the outbreak size (Δ*R*) as a function of the fraction of initially infected nodes. The red curve indicates the relative performance difference of the community-aware ranking strategy with the descending order ranking for the six centrality measures under test. The community structure is identified by the Infomap community detection algorithm.
(PNG)

S13 Fig Impact of the community structure strength (*μ*) in real-world networks. The figures represent the relative difference of the outbreak size (Δ*R*) as a function of the fraction of initially infected nodes. The red curve indicates the relative performance difference of the community-aware ranking strategy with the descending order ranking for the six centrality measures under test. The community structure is identified by the Infomap community detection algorithm.
(PNG)

S14 Fig The performance of the community-aware ranking scheme using the Louvain community detection algorithm. The figures represent the relative difference of the outbreak size (Δ*R*) as a function of the fraction of initially infected nodes. The red curve indicates the relative performance difference of the community-aware ranking strategy with the descending order ranking for the six centrality measures under test.
(PNG)

S15 Fig The performance of the community-aware ranking scheme using the Louvain community detection algorithm. The figures represent the relative difference of the outbreak size (Δ*R*) as a function of the fraction of initially infected nodes. The red curve indicates the relative performance difference of the community-aware ranking strategy with the descending order ranking for the six centrality measures under test.
(PNG)

S16 Fig The performance of the community-aware ranking scheme using the Louvain community detection algorithm. The figures represent the relative difference of the outbreak size (Δ*R*) as a function of the fraction of initially infected nodes. The red curve indicates the relative performance difference of the community-aware ranking strategy with the descending order ranking for the six centrality measures under test.
(PNG)

S17 Fig The performance of the community-aware ranking scheme using the Louvain community detection algorithm. The figures represent the relative difference of the outbreak size (Δ*R*) as a function of the fraction of initially infected nodes. The red curve indicates the relative performance difference of the community-aware ranking strategy with the descending order ranking for the six centrality measures under test.
(PNG)

S18 Fig The performance of the community-aware ranking scheme using the Louvain community detection algorithm. The figures represent the relative difference of the outbreak size (Δ*R*) as a function of the fraction of initially infected nodes. The red curve indicates the relative performance difference of the community-aware ranking strategy with the descending order ranking for the six centrality measures under test.
(PNG)

S19 Fig The performance of the community-aware ranking scheme using the Louvain community detection algorithm. The figures represent the relative difference of the outbreak size (Δ*R*) as a function of the fraction of initially infected nodes. The red curve indicates the relative performance difference of the community-aware ranking strategy with the descending order ranking for the six centrality measures under test.
(PNG)

S20 Fig The performance of the community-aware ranking scheme using the Louvain community detection algorithm. The figures represent the relative difference of the outbreak size (Δ*R*) as a function of the fraction of initially infected nodes. The red curve indicates the relative performance difference of the community-aware ranking strategy with the descending order ranking for the six centrality measures under test.
(PNG)

S21 Fig The histograms of the community size distribution for the real-world networks. Communities are identified by the Infomap (A) and Louvain (B) community detection algorithms.
(PNG)

S22 Fig The histograms of the community size distribution for the real-world networks. Communities are identified by the Infomap (A) and Louvain (B) community detection algorithms.
(PNG)

S23 Fig The histograms of the community size distribution for the real-world networks. Communities are identified by the Infomap (A) and Louvain (B) community detection algorithms.
(PNG)

S1 Text Supplementary material. (DOCX)
