# Supplementary figures and images for: Ranking influential nodes in complex networks with community structure
Source: PLoS One. 2022 Aug 29;17(8):e0273610. doi: 10.1371/journal.pone.0273610 (PMC9423620; doi:10.1371/journal.pone.0273610)

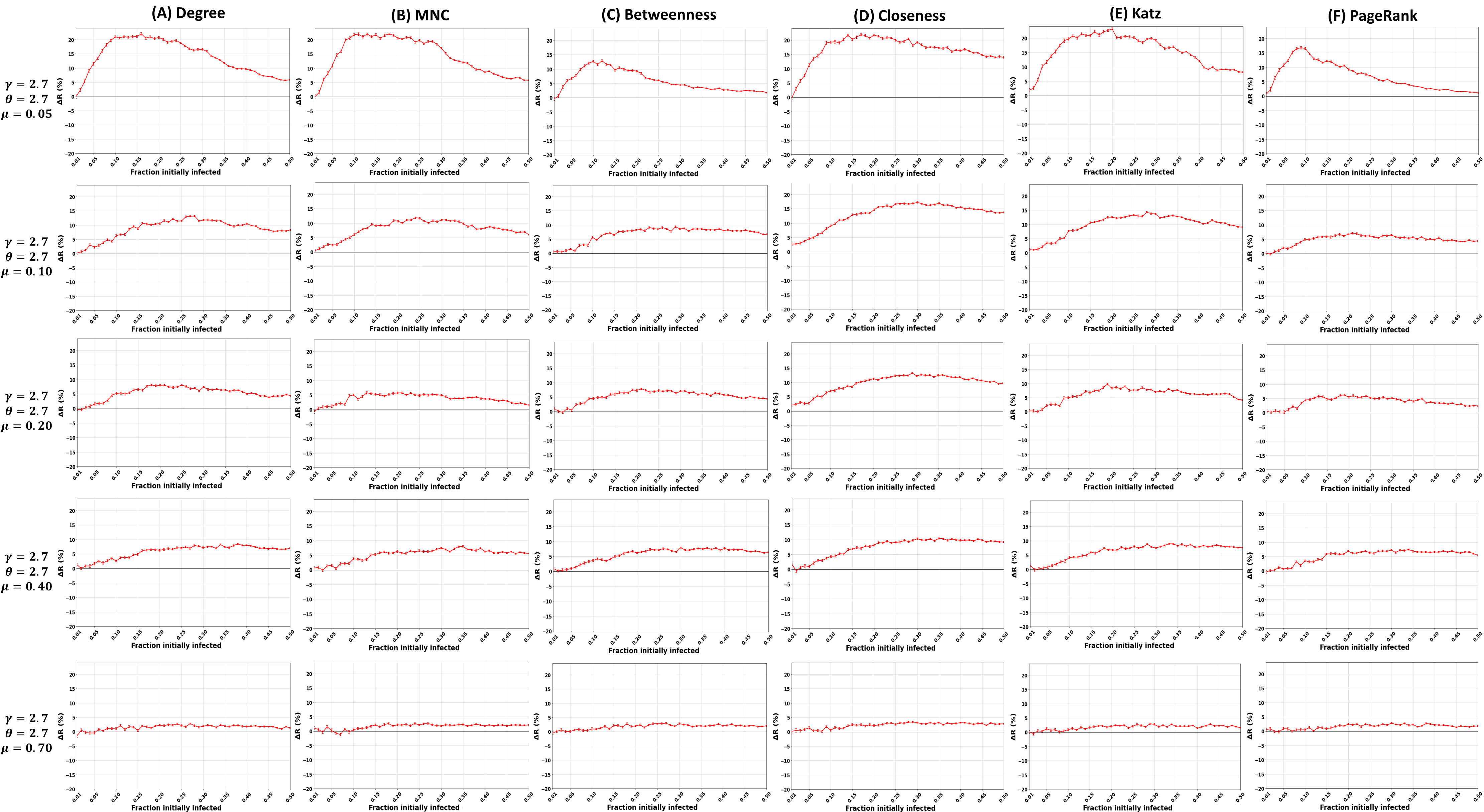

Supplement: S1 Fig — The figures represent the relative difference of the outbreak size (ΔR) as a function of the fraction of initially infected nodes. The red curve indicates the relative performance difference of the community-aware ranking strategy with the descending order ranking for the six centrality measures under test. The mixing parameter (μ) is varied while the other parameters, including the community size distribution exponent (θ = 2.7) and the degree distribution exponent (γ = 2.7), are fixed. (PNG) [file pone.0273610.s001.png]

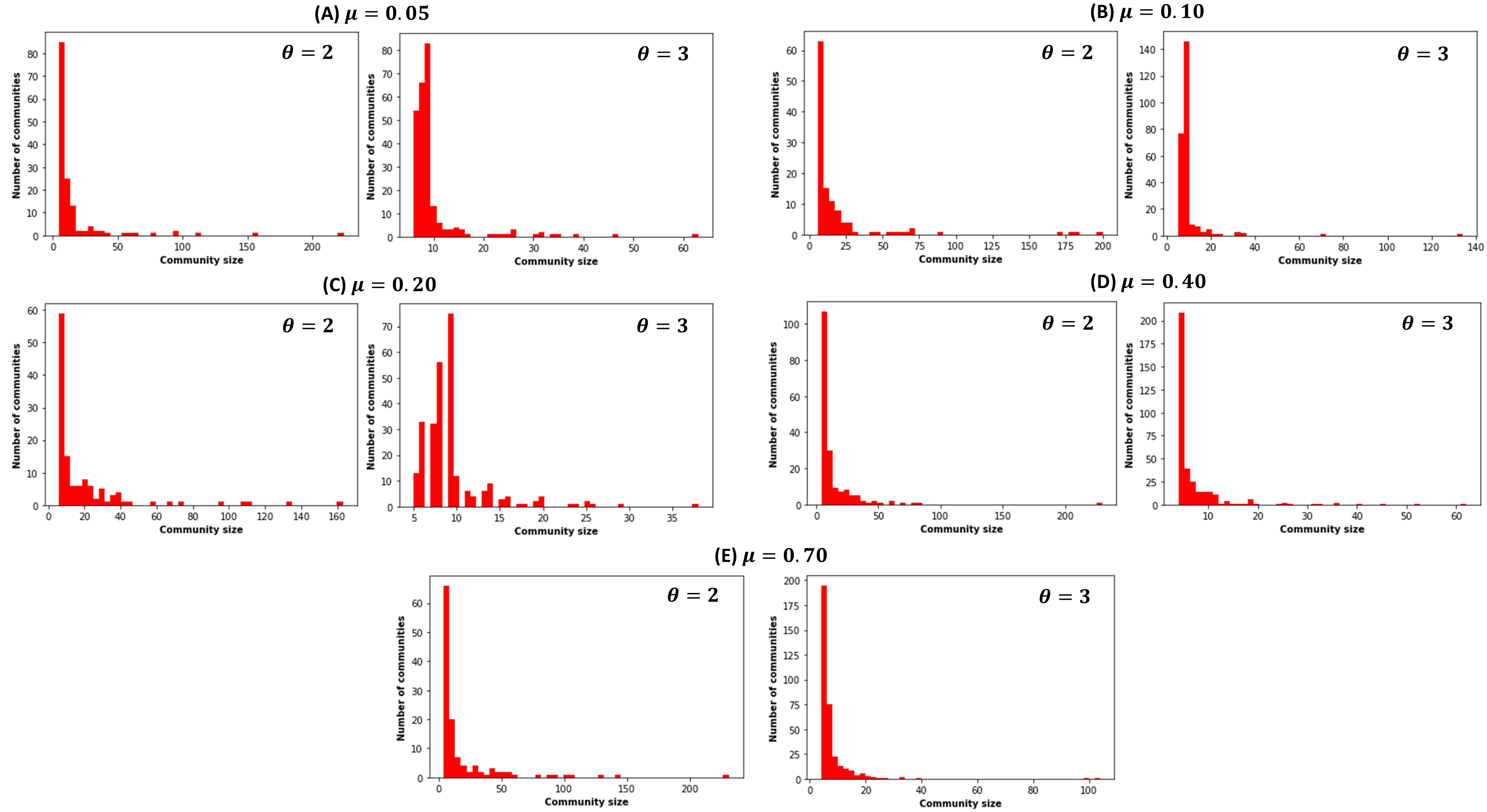

Supplement: S2 Fig — The synthetic networks are generated with θ = 2 and θ = 3 from strong to weak community structure strengths while keeping other parameters fixed including the degree distribution exponent (γ = 2.7). (PNG) [file pone.0273610.s002.png]

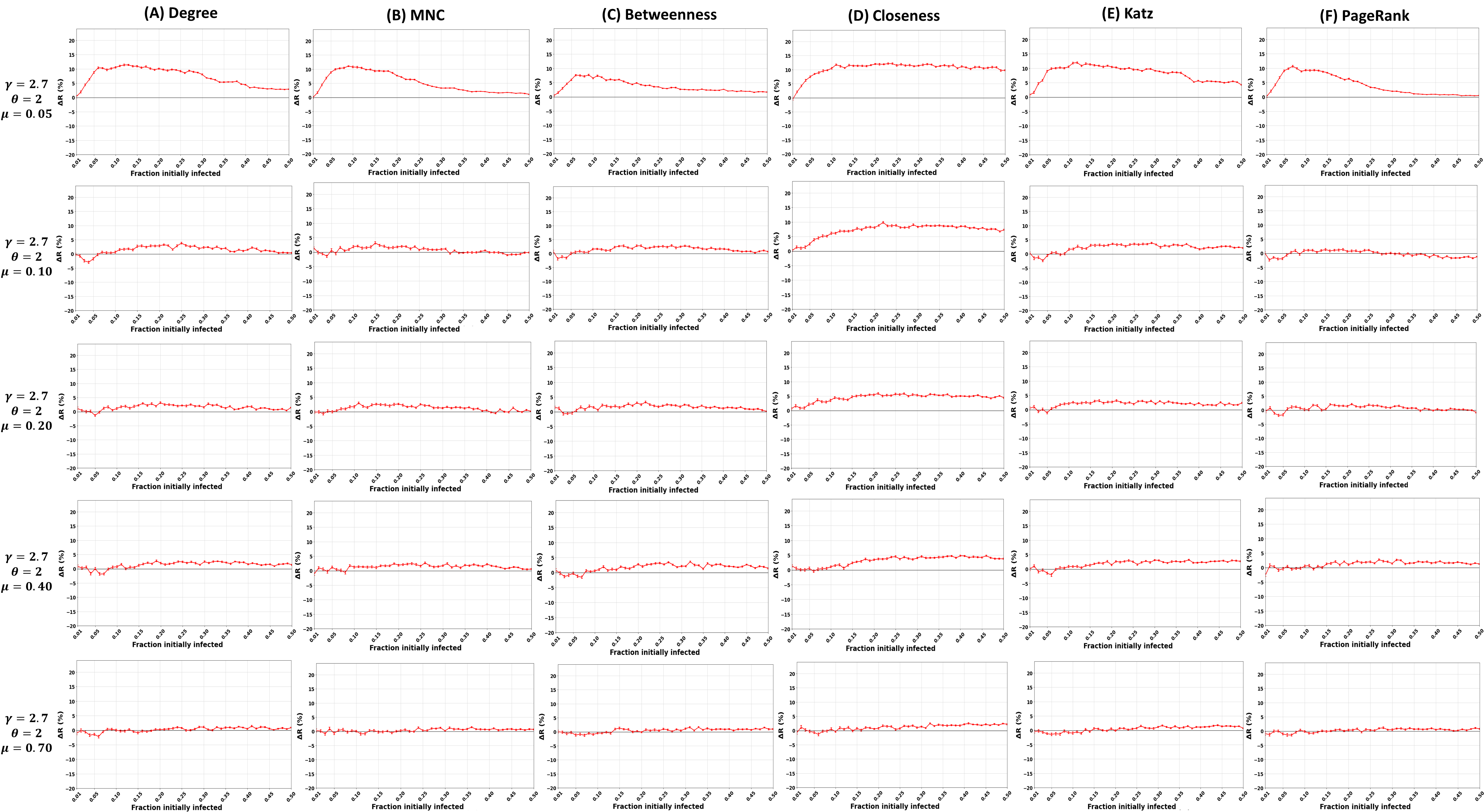

Supplement: S3 Fig — The figures represent the relative difference of the outbreak size (ΔR) as a function of the fraction of initially infected nodes. The red curve indicates the relative performance difference of the community-aware ranking strategy with the descending order ranking for the six centrality measures under test. The mixing parameter (μ) is varied while the other parameters, including the community size distribution exponent (θ = 2) and the degree distribution exponent (γ = 2.7), are fixed. (PNG) [file pone.0273610.s003.png]

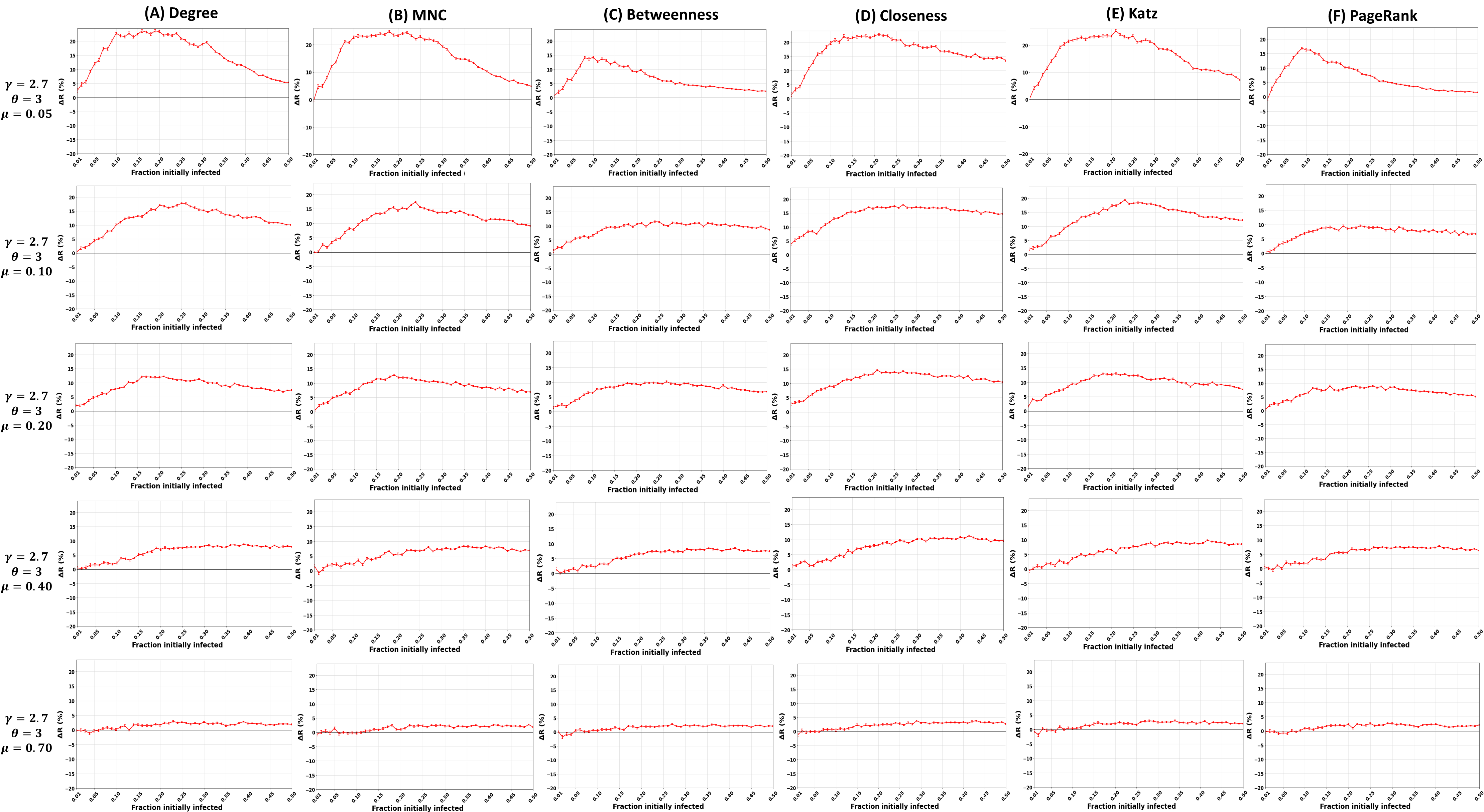

Supplement: S4 Fig — The figures represent the relative difference of the outbreak size (ΔR) as a function of the fraction of initially infected nodes. The red curve indicates the relative performance difference of the community-aware ranking strategy with the descending order ranking for the six centrality measures under test. The mixing parameter (μ) is varied while the other parameters, including the community size distribution exponent (θ = 3) and the degree distribution exponent (γ = 2.7), are fixed. (PNG) [file pone.0273610.s004.png]

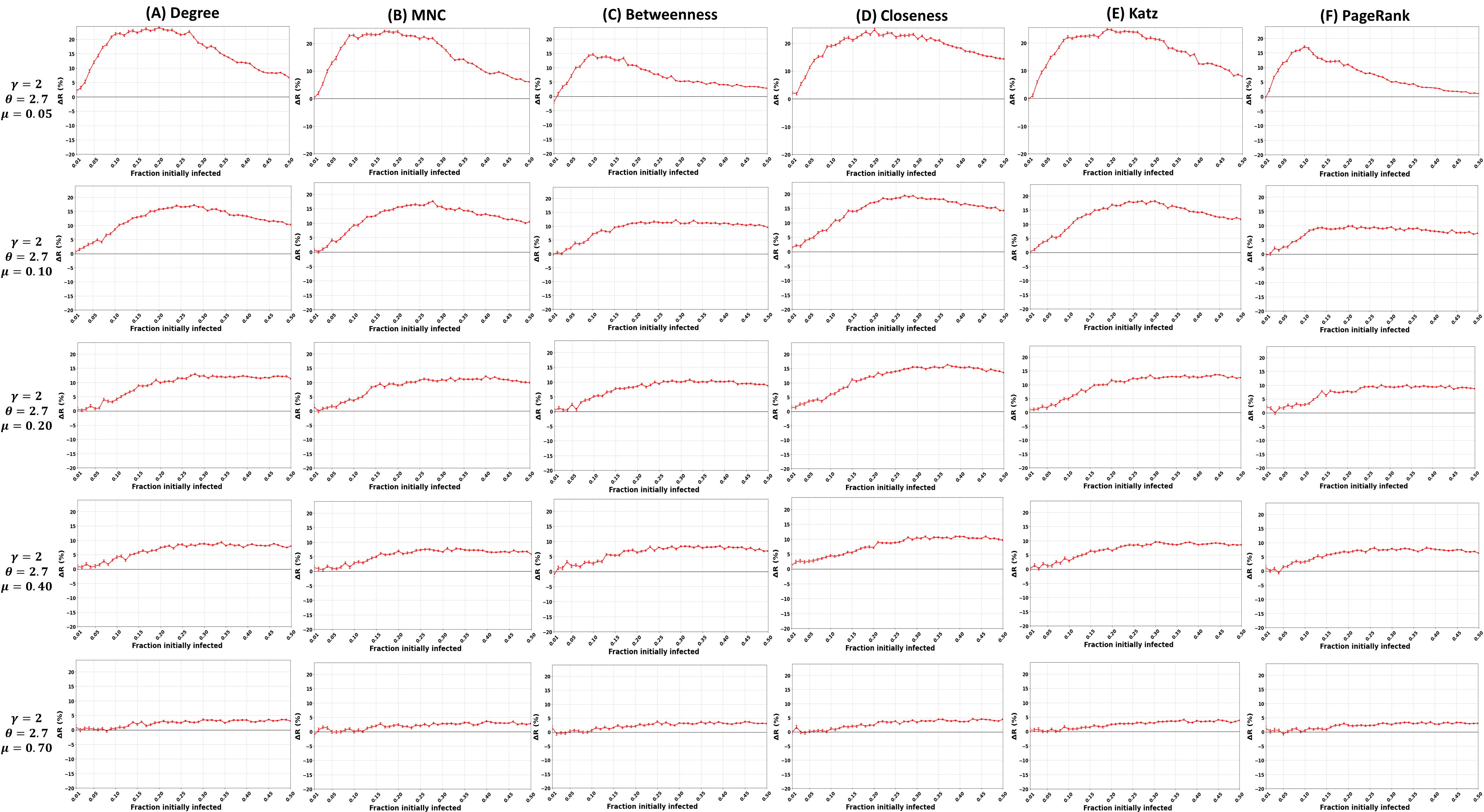

Supplement: S5 Fig — The figures represent the relative difference of the outbreak size (ΔR) as a function of the fraction of initially infected nodes. The red curve indicates the relative performance difference of the community-aware ranking strategy with the descending order ranking for the six centrality measures under test. The mixing parameter (μ) is varied while the other parameters, including the community size distribution exponent (θ = 2.7) and the degree distribution exponent (γ = 2), are fixed. (PNG) [file pone.0273610.s005.png]

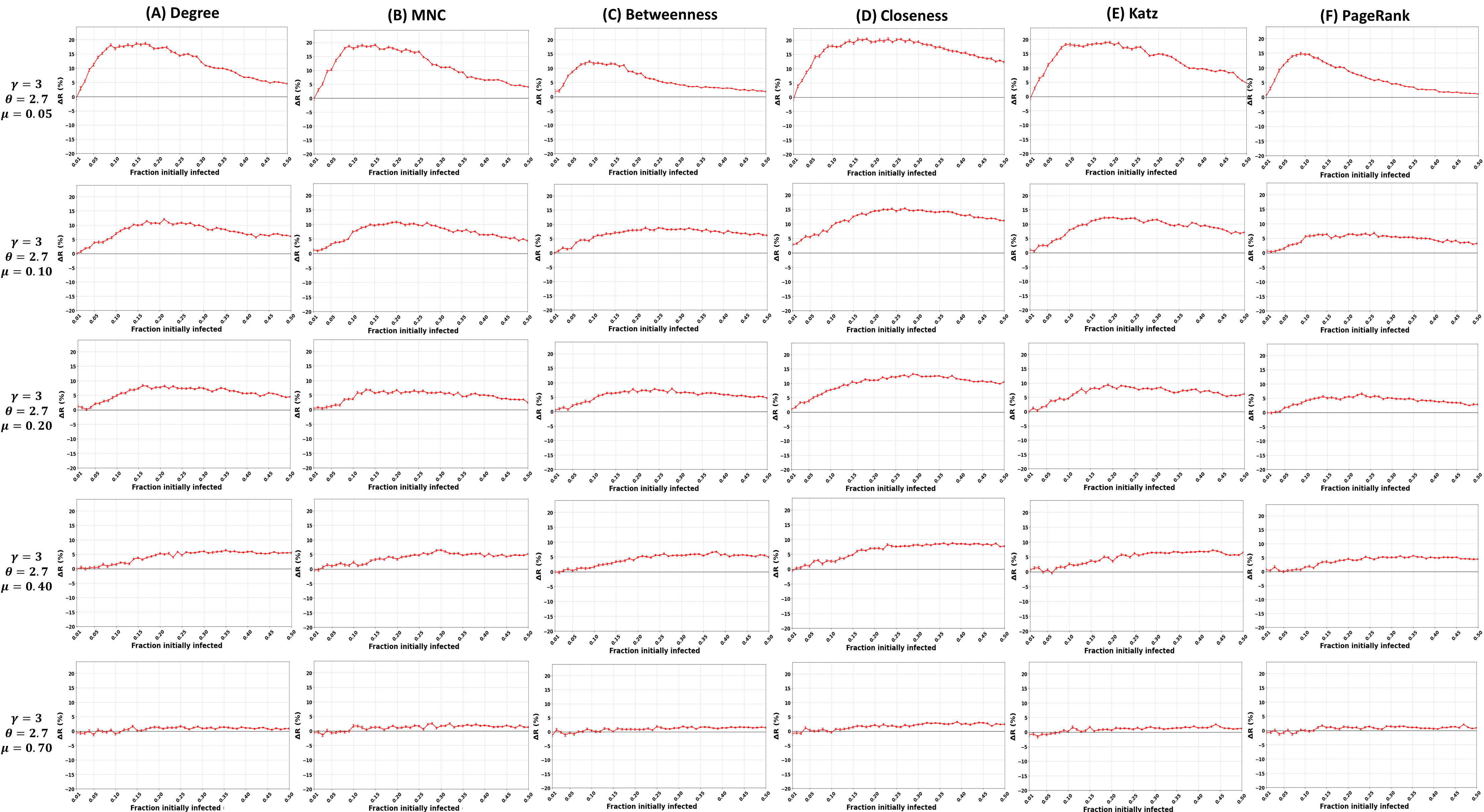

Supplement: S6 Fig — The figures represent the relative difference of the outbreak size (ΔR) as a function of the fraction of initially infected nodes. The red curve indicates the relative performance difference of the community-aware ranking strategy with the descending order ranking for the six centrality measures under test. The mixing parameter (μ) is varied while the other parameters, including the community size distribution exponent (θ = 2.7) and the degree distribution exponent (γ = 3), are fixed. (PNG) [file pone.0273610.s006.png]

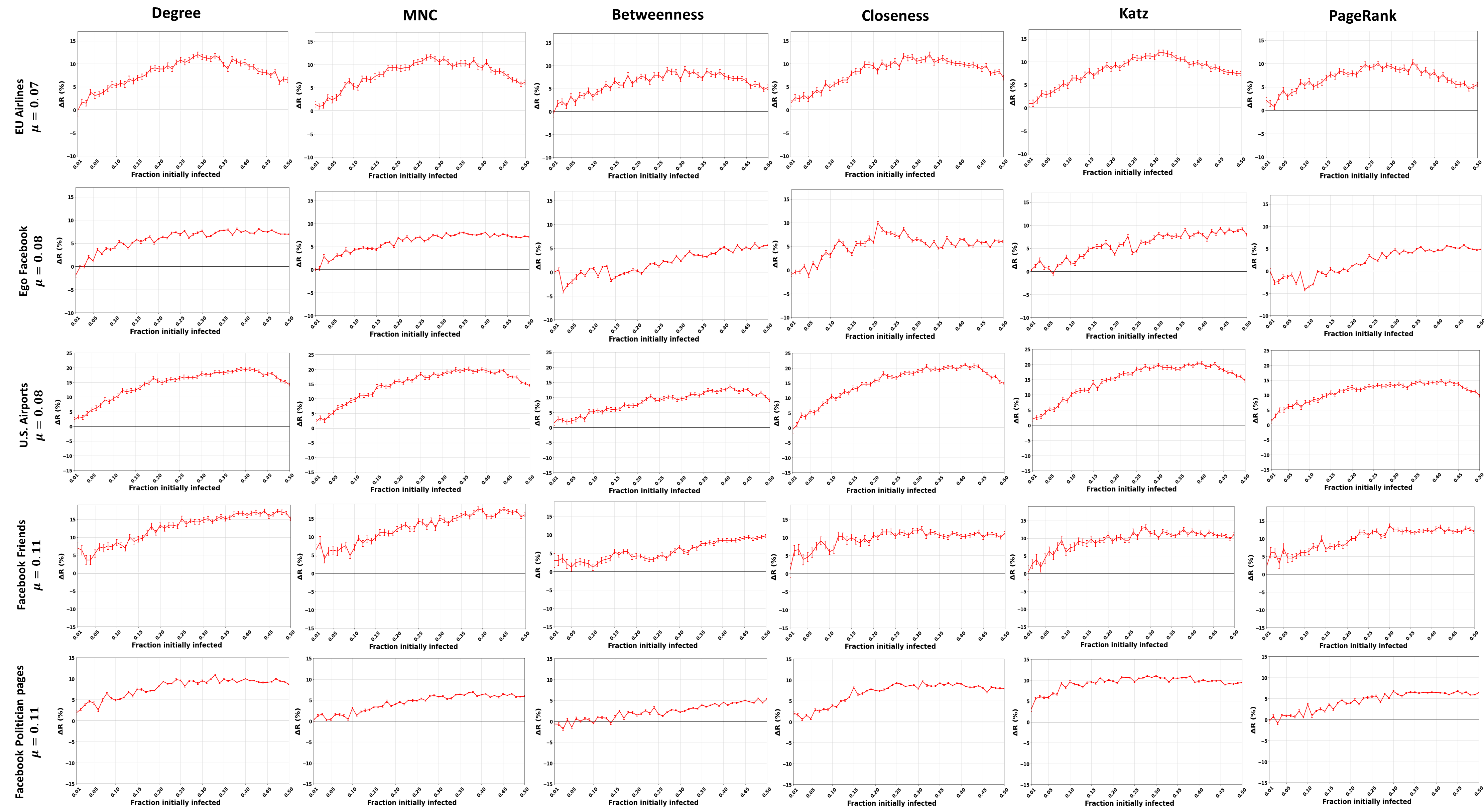

Supplement: S7 Fig — The figures represent the relative difference of the outbreak size (ΔR) as a function of the fraction of initially infected nodes. The red curve indicates the relative performance difference of the community-aware ranking strategy with the descending order ranking for the six centrality measures under test. The community structure is identified by the Infomap community detection algorithm. (PNG) [file pone.0273610.s007.png]

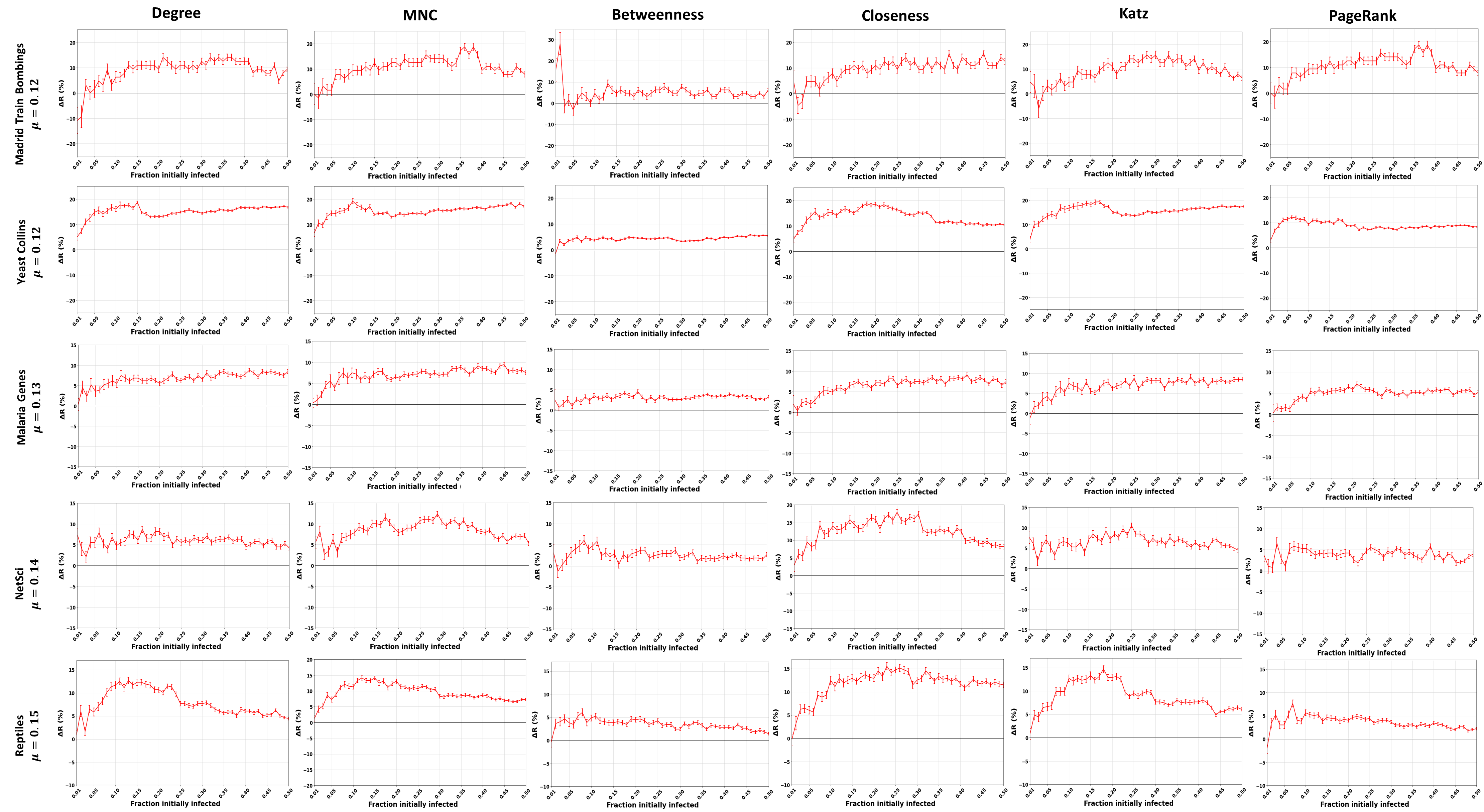

Supplement: S8 Fig — The figures represent the relative difference of the outbreak size (ΔR) as a function of the fraction of initially infected nodes. The red curve indicates the relative performance difference of the community-aware ranking strategy with the descending order ranking for the six centrality measures under test. The community structure is identified by the Infomap community detection algorithm. (PNG) [file pone.0273610.s008.png]

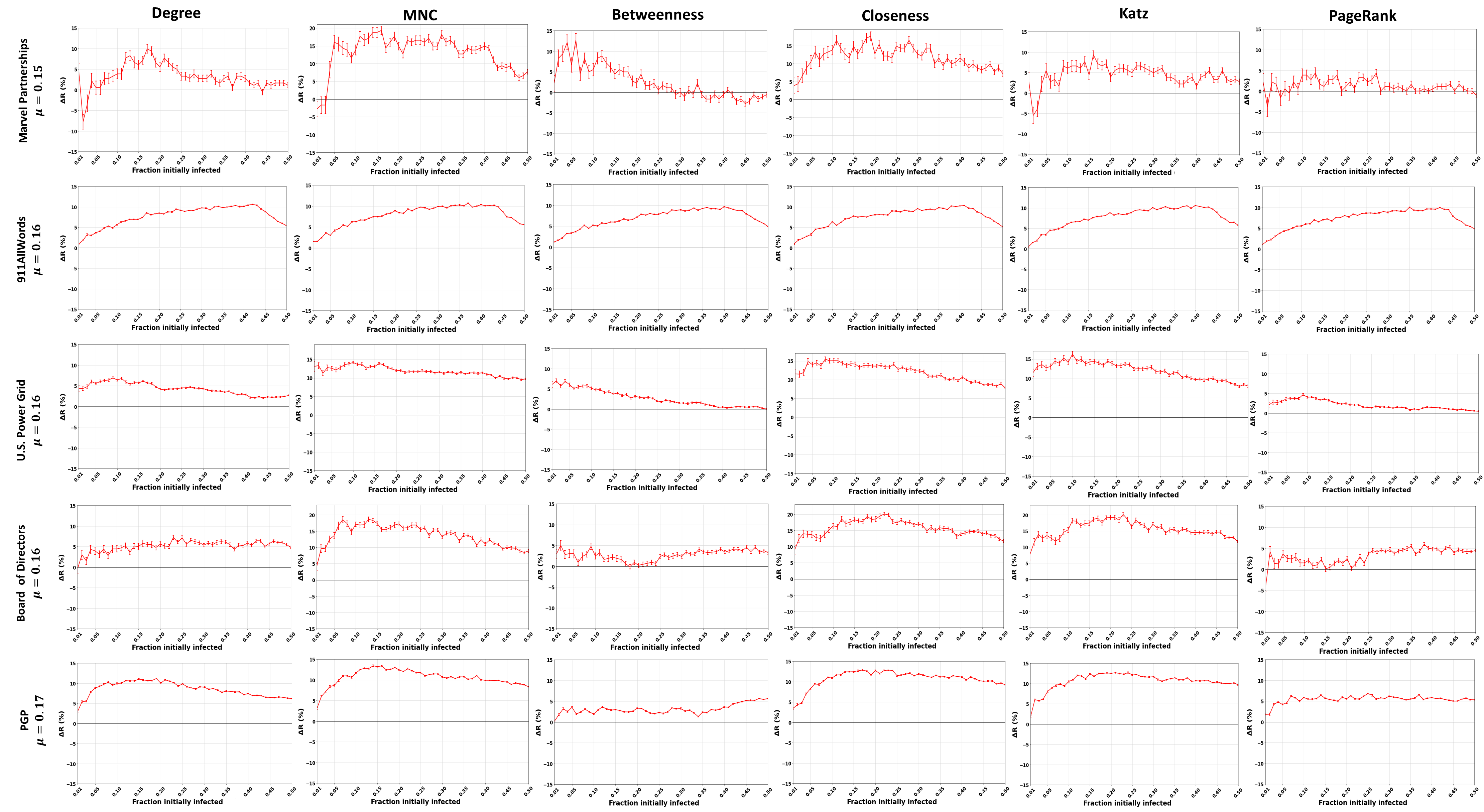

Supplement: S9 Fig — The figures represent the relative difference of the outbreak size (ΔR) as a function of the fraction of initially infected nodes. The red curve indicates the relative performance difference of the community-aware ranking strategy with the descending order ranking for the six centrality measures under test. The community structure is identified by the Infomap community detection algorithm. (PNG) [file pone.0273610.s009.png]

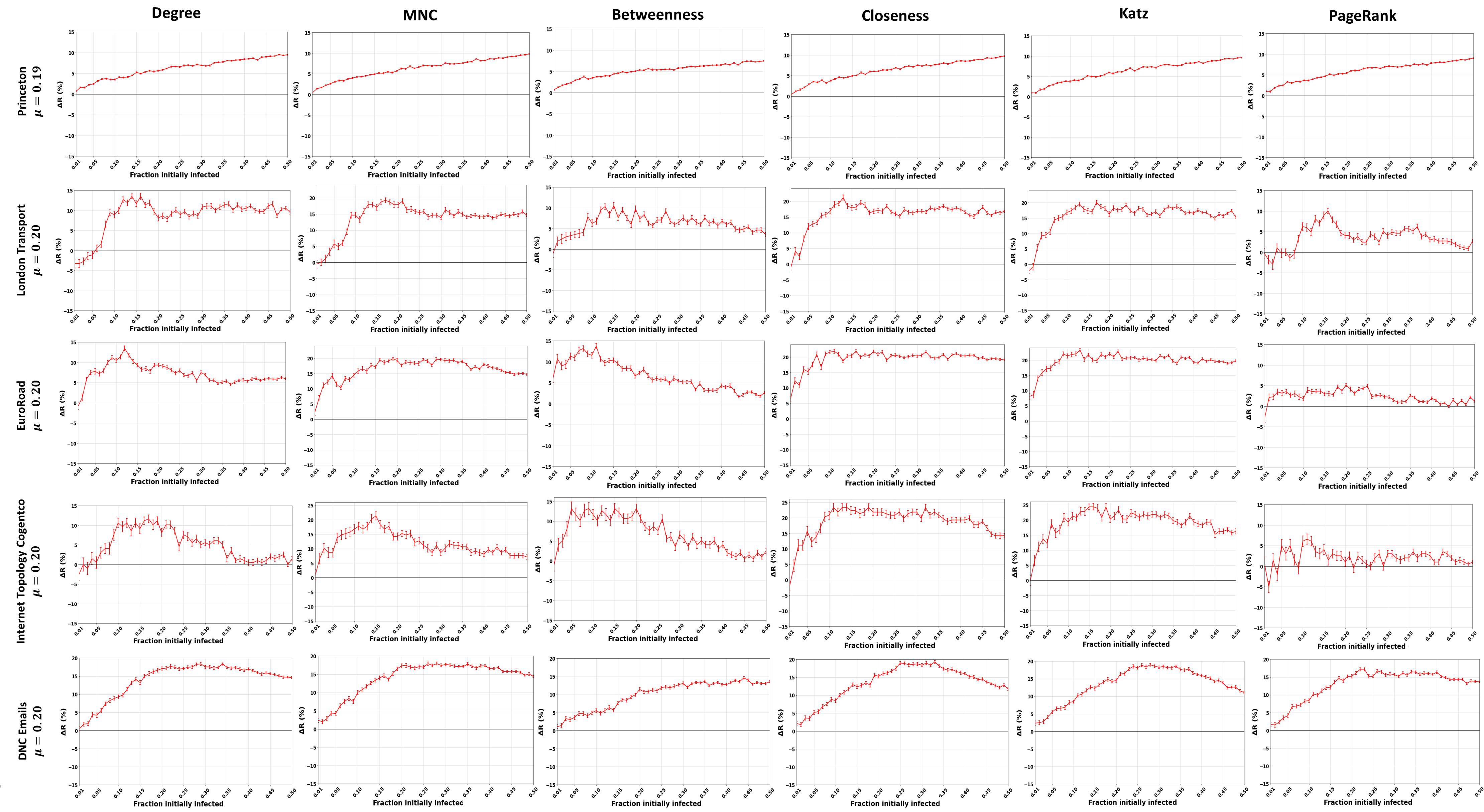

Supplement: S10 Fig — The figures represent the relative difference of the outbreak size (ΔR) as a function of the fraction of initially infected nodes. The red curve indicates the relative performance difference of the community-aware ranking strategy with the descending order ranking for the six centrality measures under test. The community structure is identified by the Infomap community detection algorithm. (PNG) [file pone.0273610.s010.png]

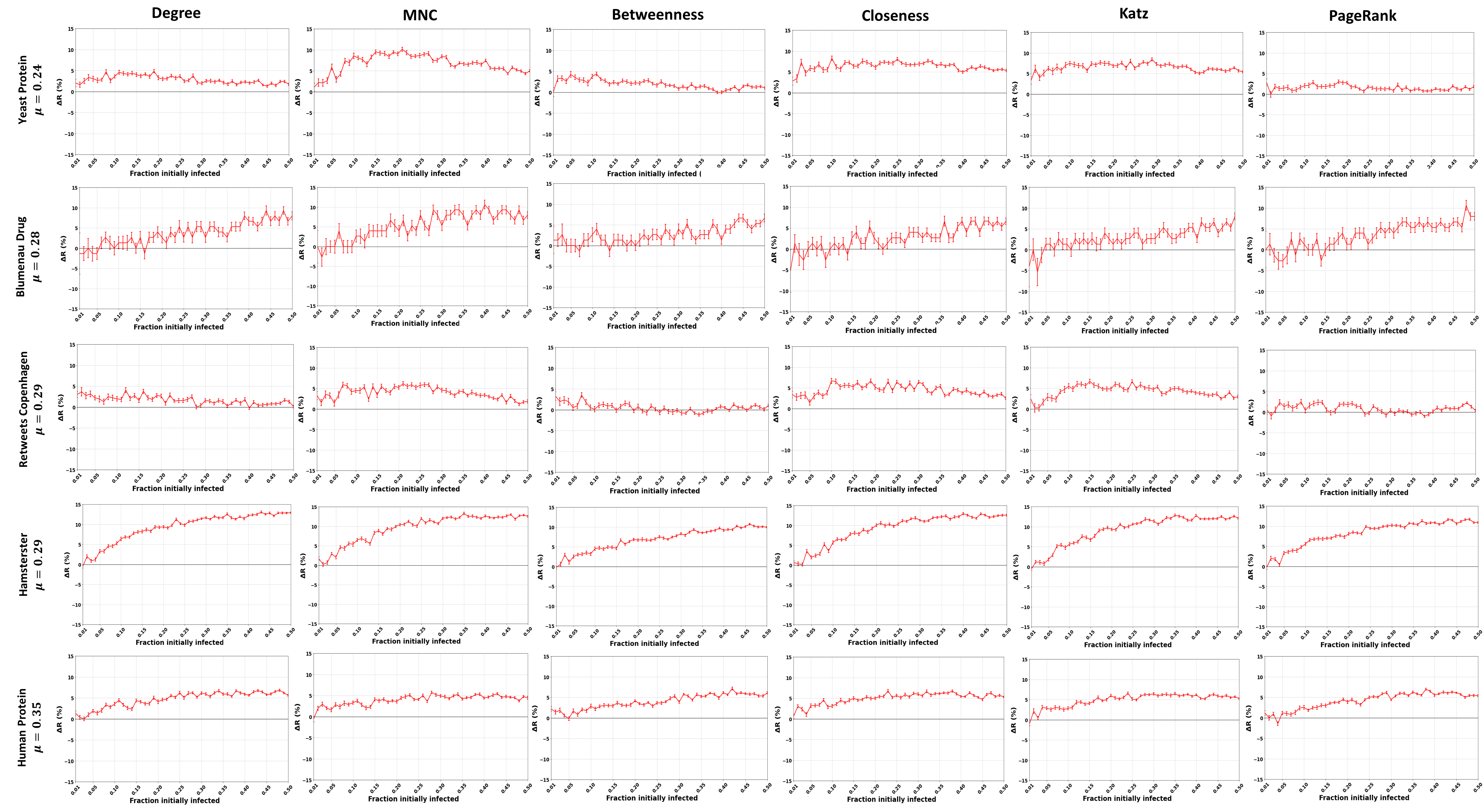

Supplement: S11 Fig — The figures represent the relative difference of the outbreak size (ΔR) as a function of the fraction of initially infected nodes. The red curve indicates the relative performance difference of the community-aware ranking strategy with the descending order ranking for the six centrality measures under test. The community structure is identified by the Infomap community detection algorithm. (PNG) [file pone.0273610.s011.png]

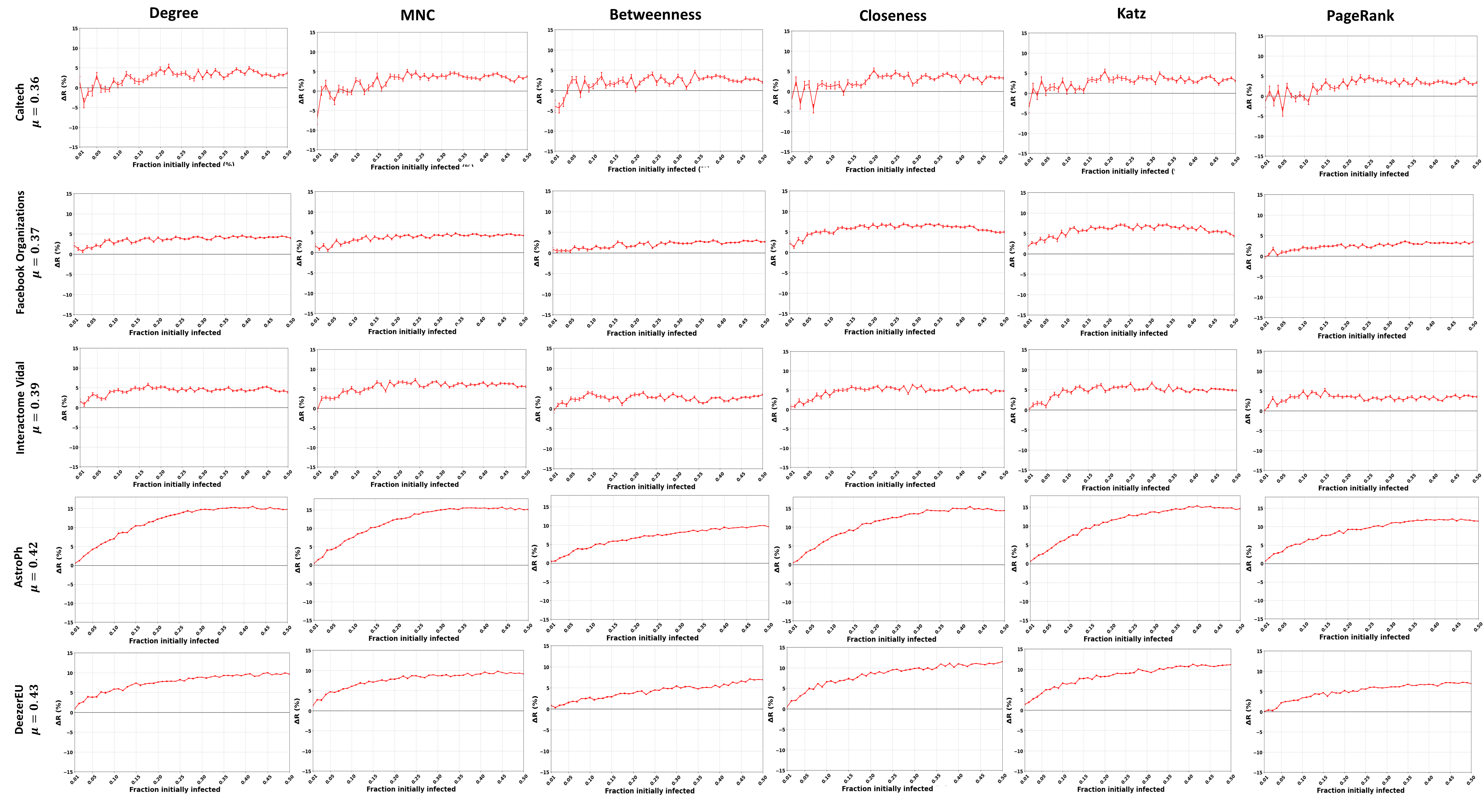

Supplement: S12 Fig — The figures represent the relative difference of the outbreak size (ΔR) as a function of the fraction of initially infected nodes. The red curve indicates the relative performance difference of the community-aware ranking strategy with the descending order ranking for the six centrality measures under test. The community structure is identified by the Infomap community detection algorithm. (PNG) [file pone.0273610.s012.png]

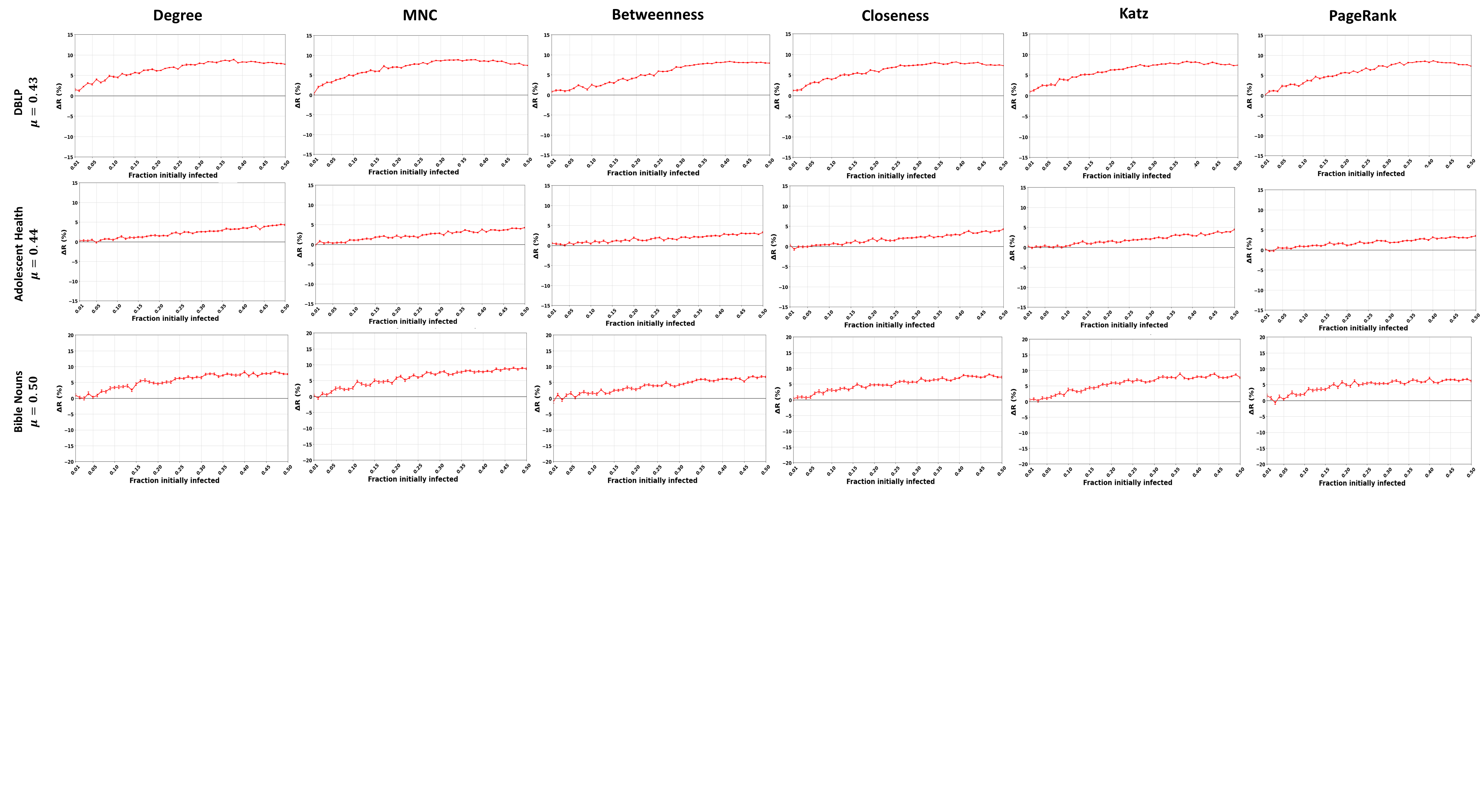

Supplement: S13 Fig — The figures represent the relative difference of the outbreak size (ΔR) as a function of the fraction of initially infected nodes. The red curve indicates the relative performance difference of the community-aware ranking strategy with the descending order ranking for the six centrality measures under test. The community structure is identified by the Infomap community detection algorithm. (PNG) [file pone.0273610.s013.png]

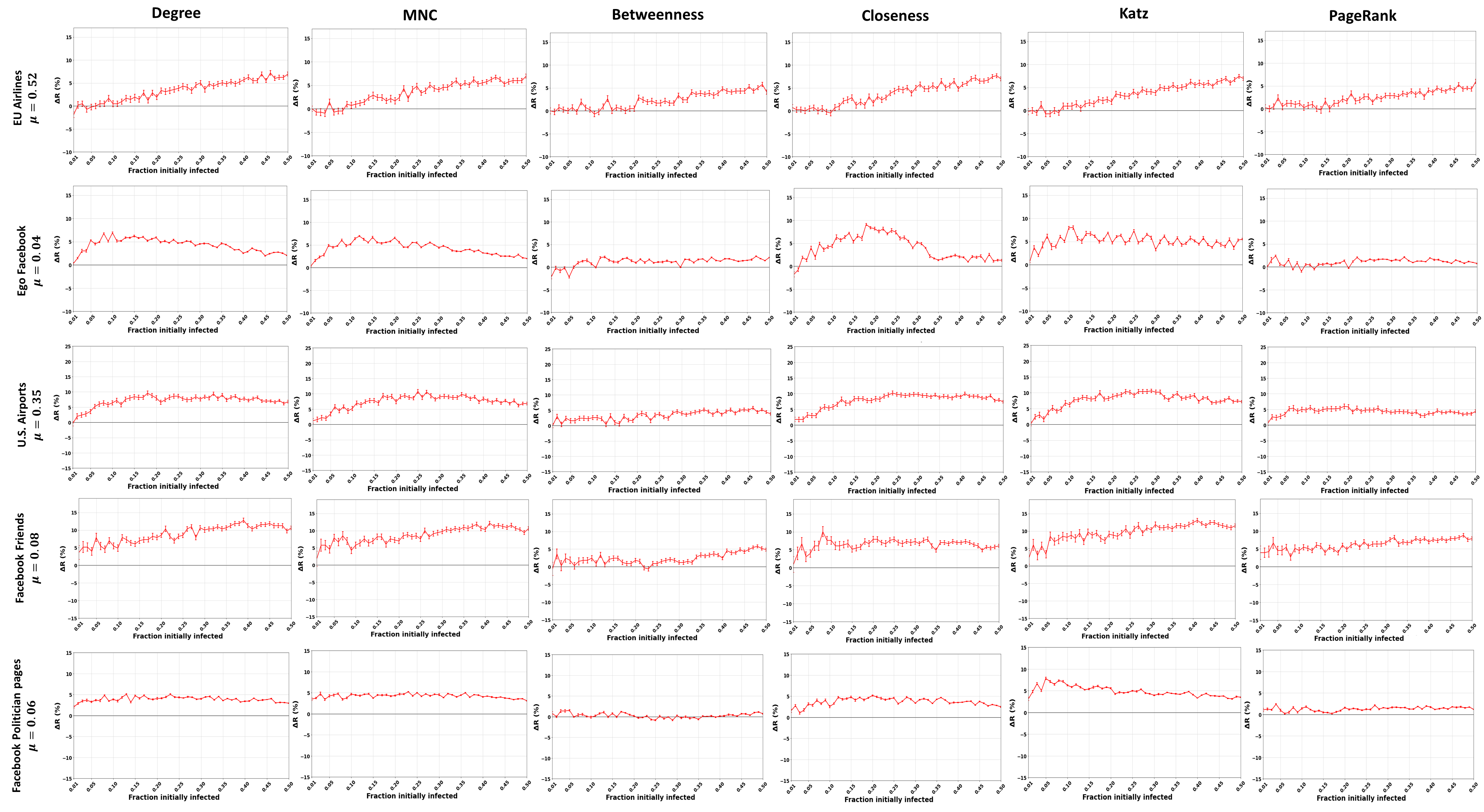

Supplement: S14 Fig — The figures represent the relative difference of the outbreak size (ΔR) as a function of the fraction of initially infected nodes. The red curve indicates the relative performance difference of the community-aware ranking strategy with the descending order ranking for the six centrality measures under test. (PNG) [file pone.0273610.s014.png]

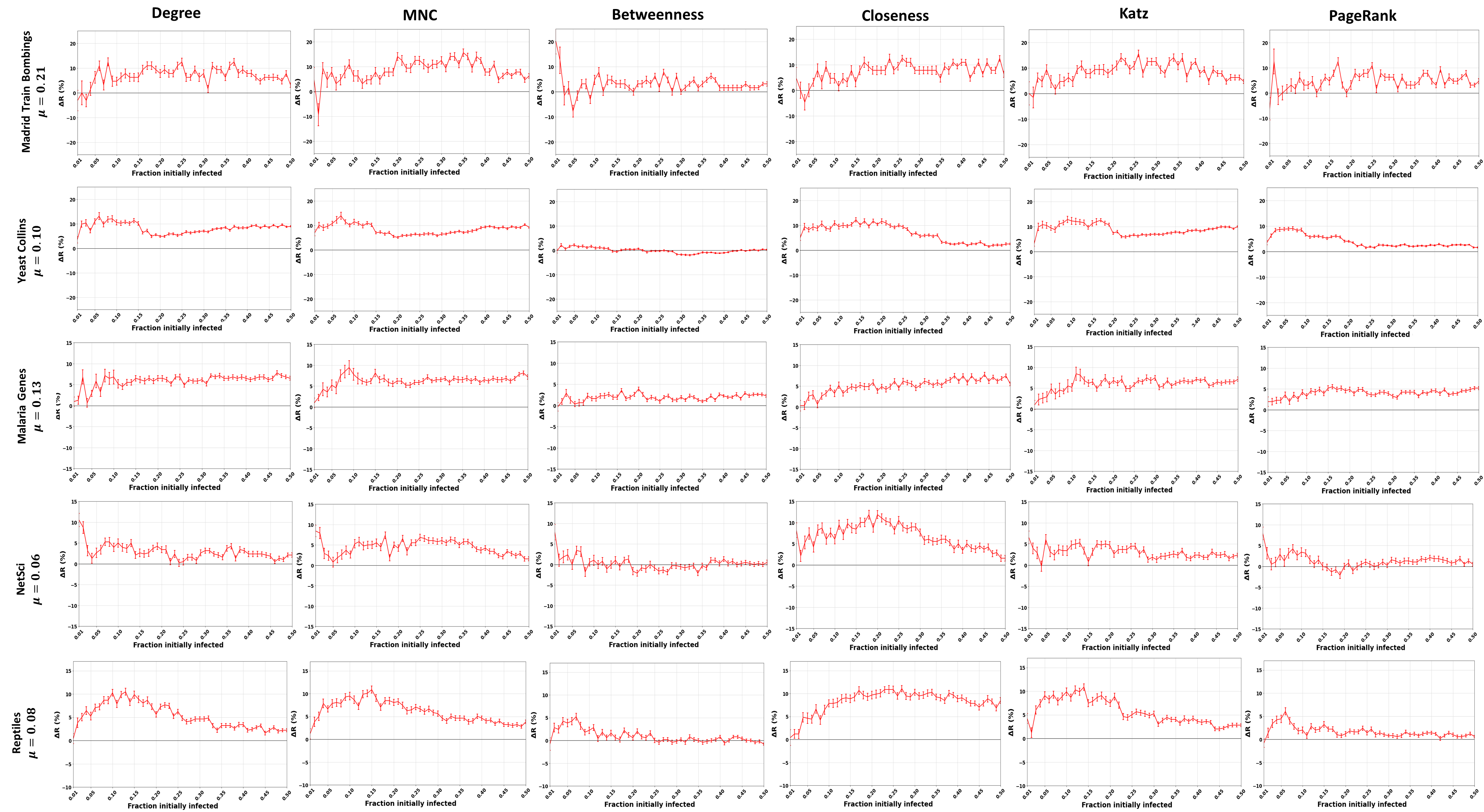

Supplement: S15 Fig — The figures represent the relative difference of the outbreak size (ΔR) as a function of the fraction of initially infected nodes. The red curve indicates the relative performance difference of the community-aware ranking strategy with the descending order ranking for the six centrality measures under test. (PNG) [file pone.0273610.s015.png]

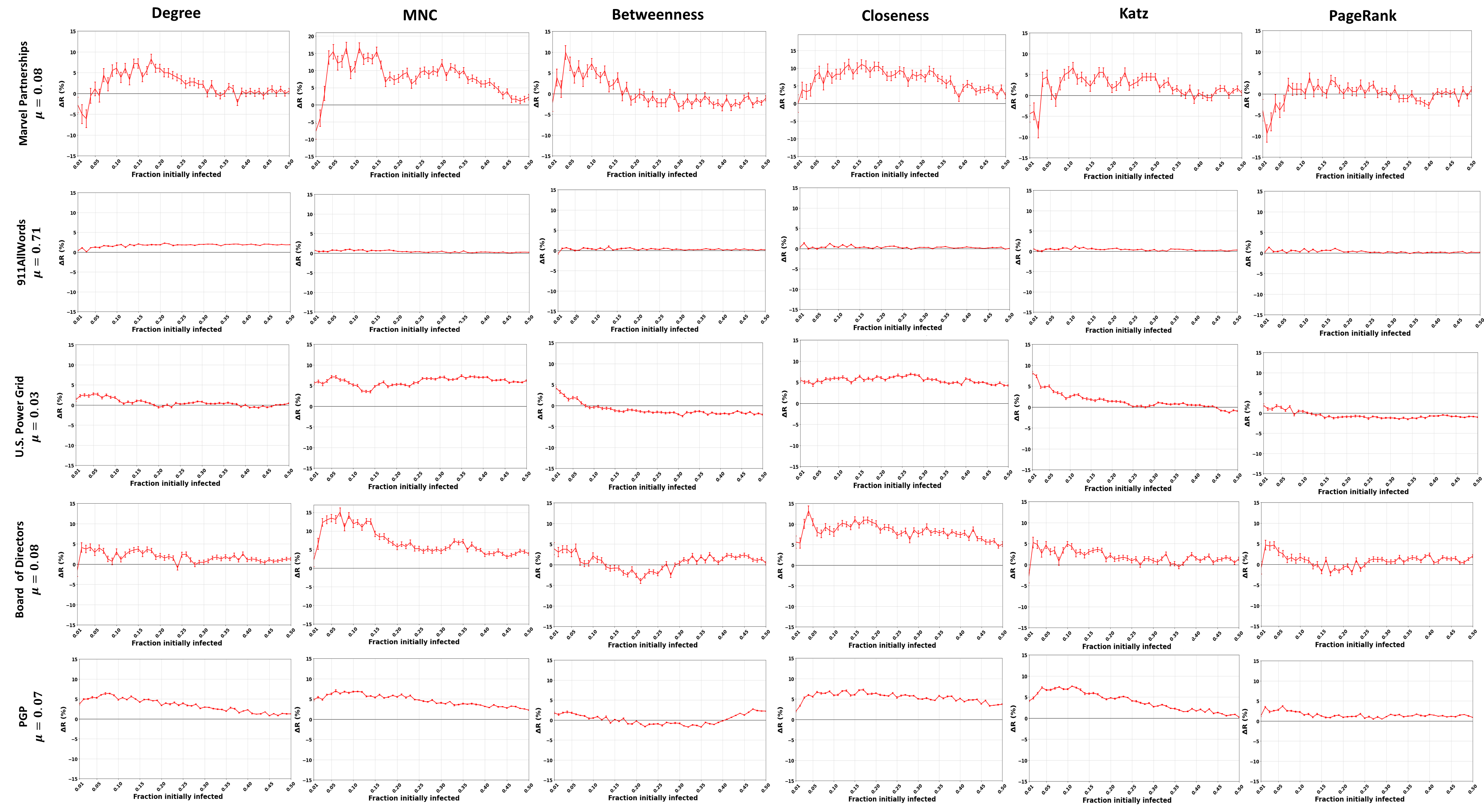

Supplement: S16 Fig — The figures represent the relative difference of the outbreak size (ΔR) as a function of the fraction of initially infected nodes. The red curve indicates the relative performance difference of the community-aware ranking strategy with the descending order ranking for the six centrality measures under test. (PNG) [file pone.0273610.s016.png]

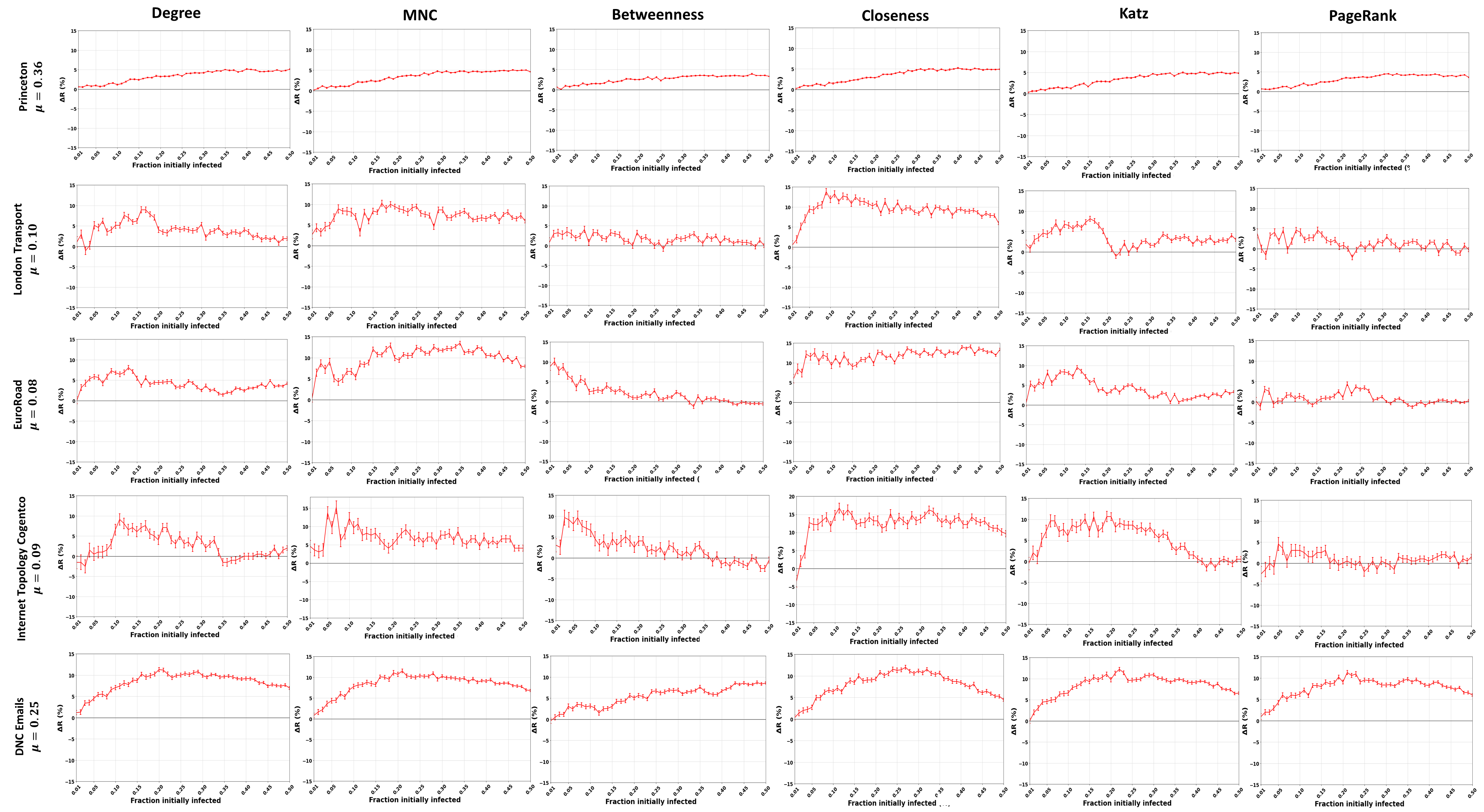

Supplement: S17 Fig — The figures represent the relative difference of the outbreak size (ΔR) as a function of the fraction of initially infected nodes. The red curve indicates the relative performance difference of the community-aware ranking strategy with the descending order ranking for the six centrality measures under test. (PNG) [file pone.0273610.s017.png]

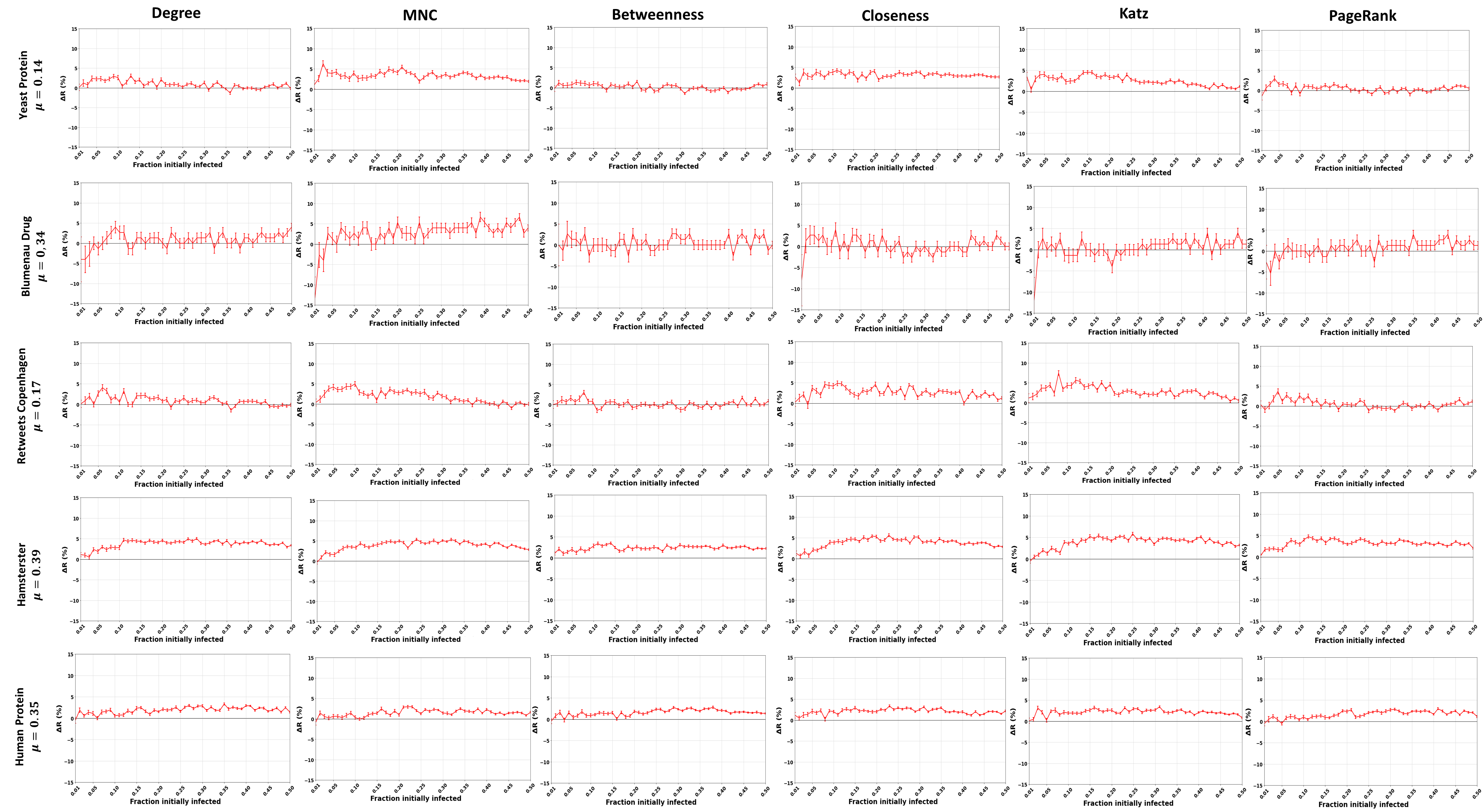

Supplement: S18 Fig — The figures represent the relative difference of the outbreak size (ΔR) as a function of the fraction of initially infected nodes. The red curve indicates the relative performance difference of the community-aware ranking strategy with the descending order ranking for the six centrality measures under test. (PNG) [file pone.0273610.s018.png]

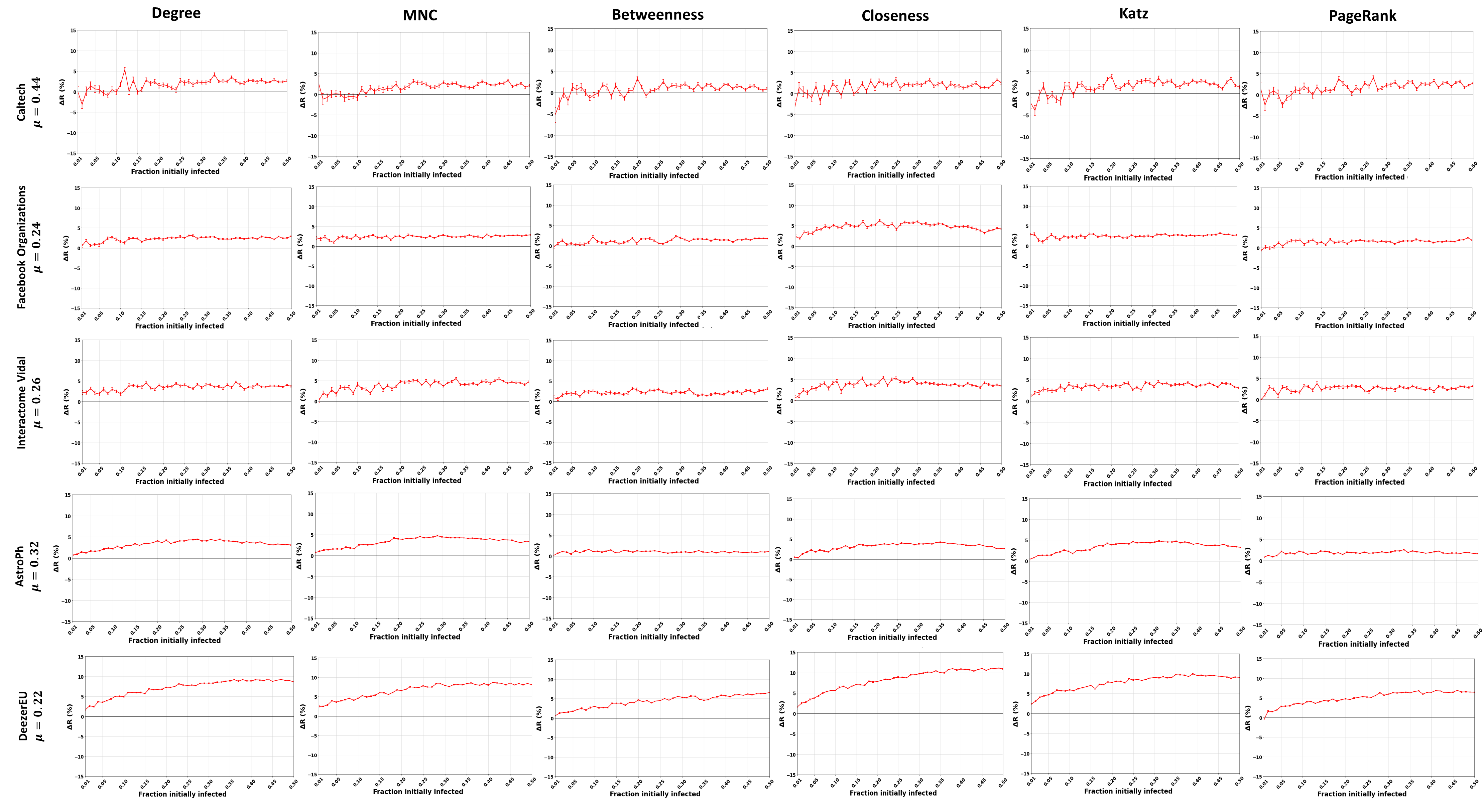

Supplement: S19 Fig — The figures represent the relative difference of the outbreak size (ΔR) as a function of the fraction of initially infected nodes. The red curve indicates the relative performance difference of the community-aware ranking strategy with the descending order ranking for the six centrality measures under test. (PNG) [file pone.0273610.s019.png]

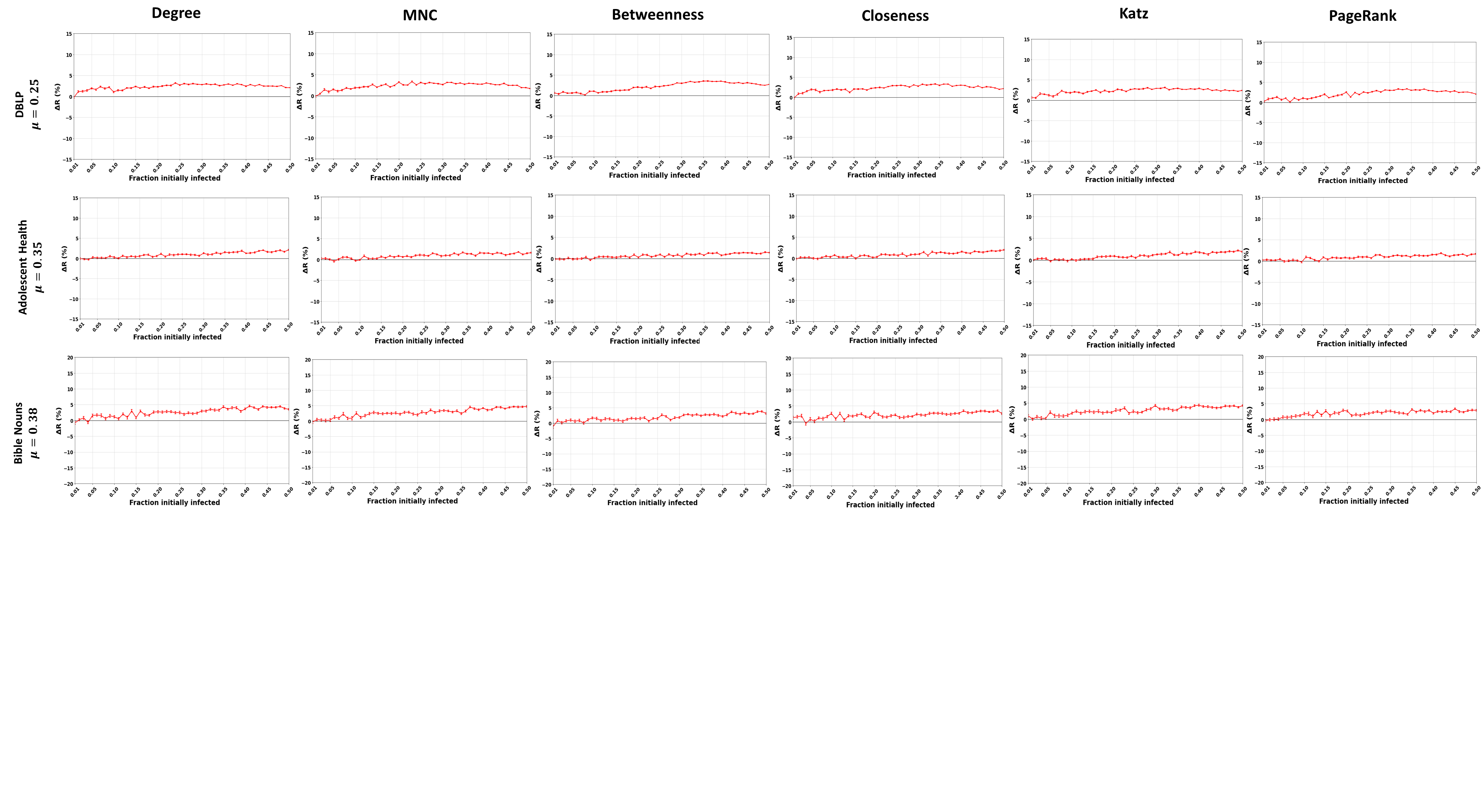

Supplement: S20 Fig — The figures represent the relative difference of the outbreak size (ΔR) as a function of the fraction of initially infected nodes. The red curve indicates the relative performance difference of the community-aware ranking strategy with the descending order ranking for the six centrality measures under test. (PNG) [file pone.0273610.s020.png]

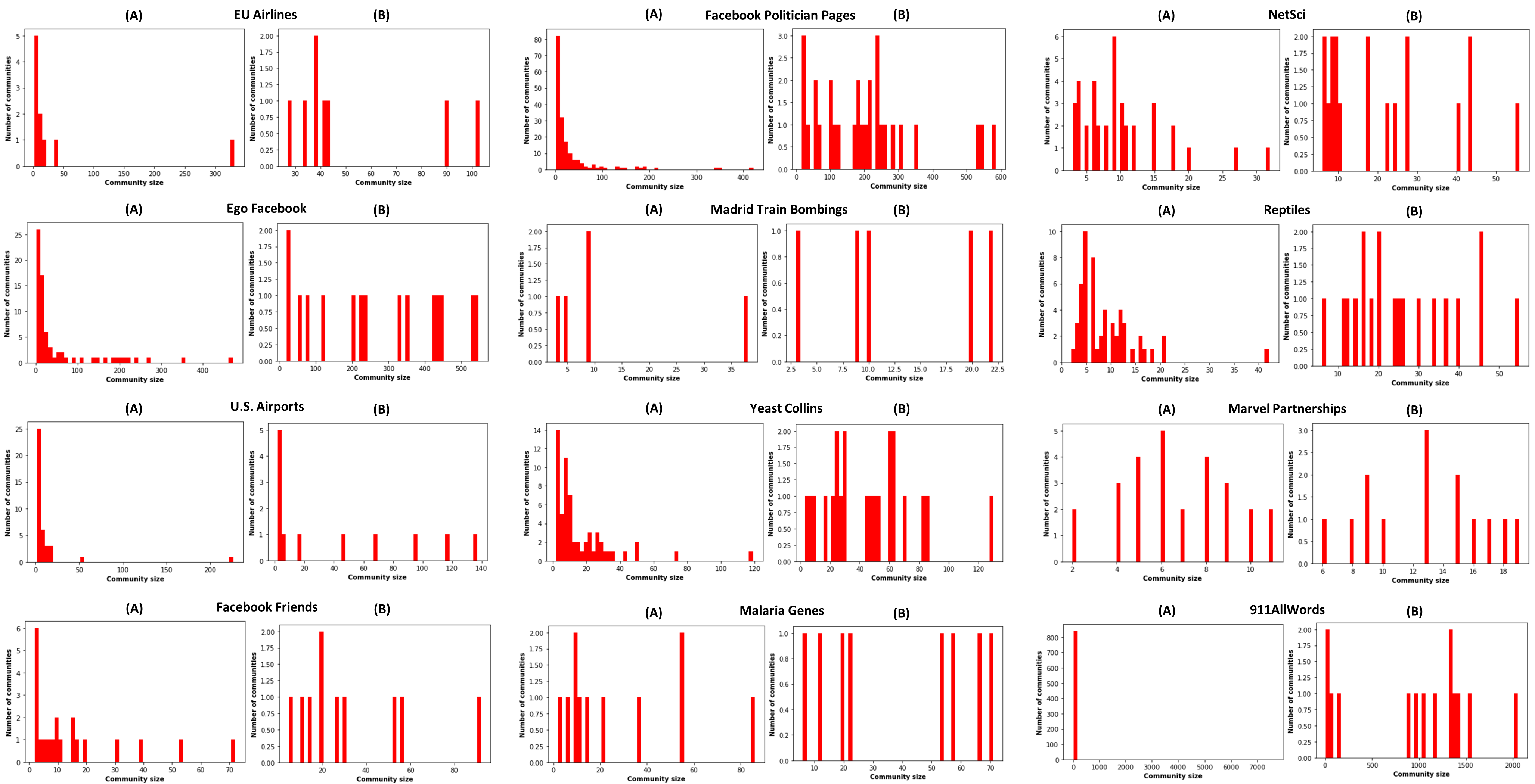

Supplement: S21 Fig — Communities are identified by the Infomap (A) and Louvain (B) community detection algorithms. (PNG) [file pone.0273610.s021.png]

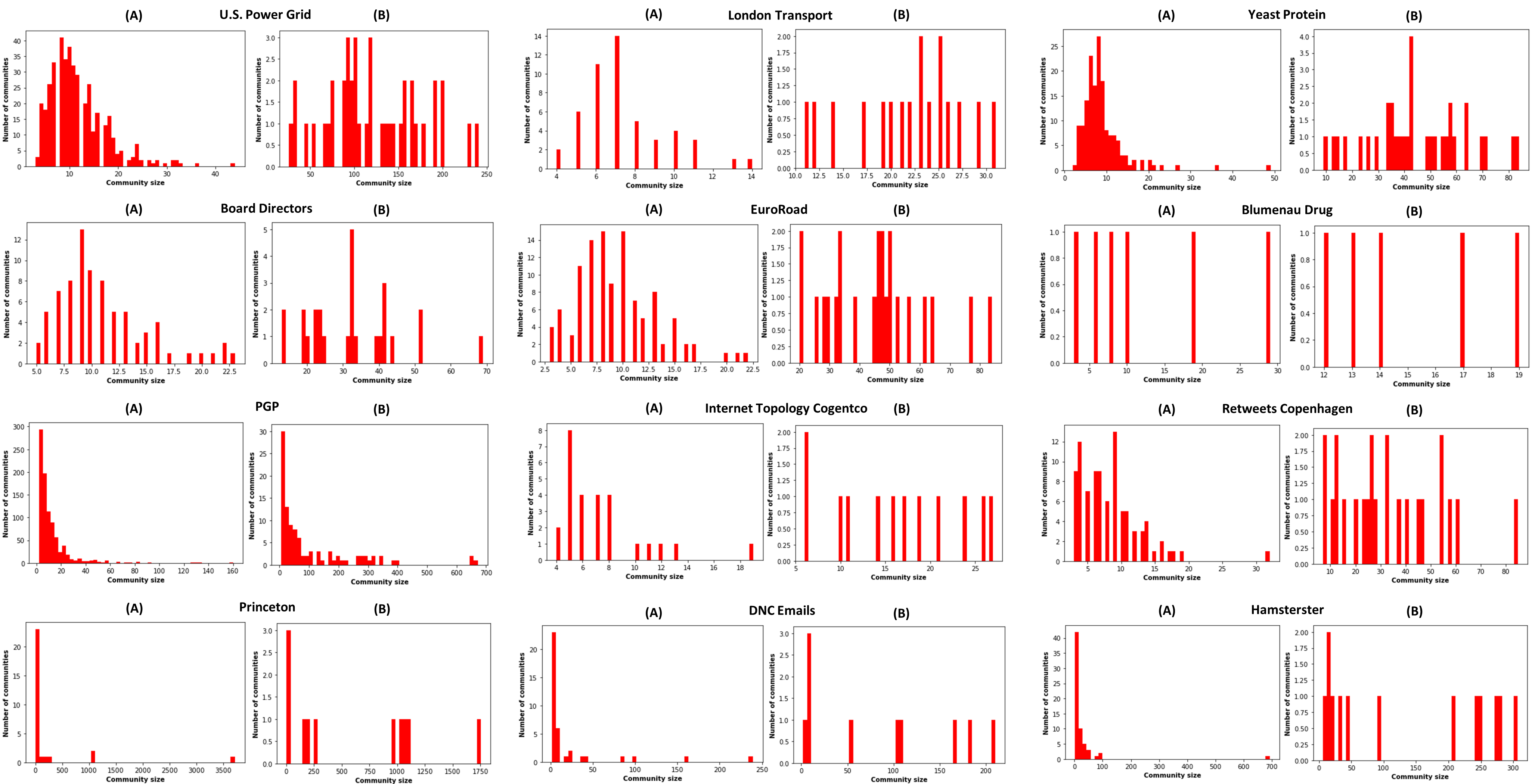

Supplement: S22 Fig — Communities are identified by the Infomap (A) and Louvain (B) community detection algorithms. (PNG) [file pone.0273610.s022.png]

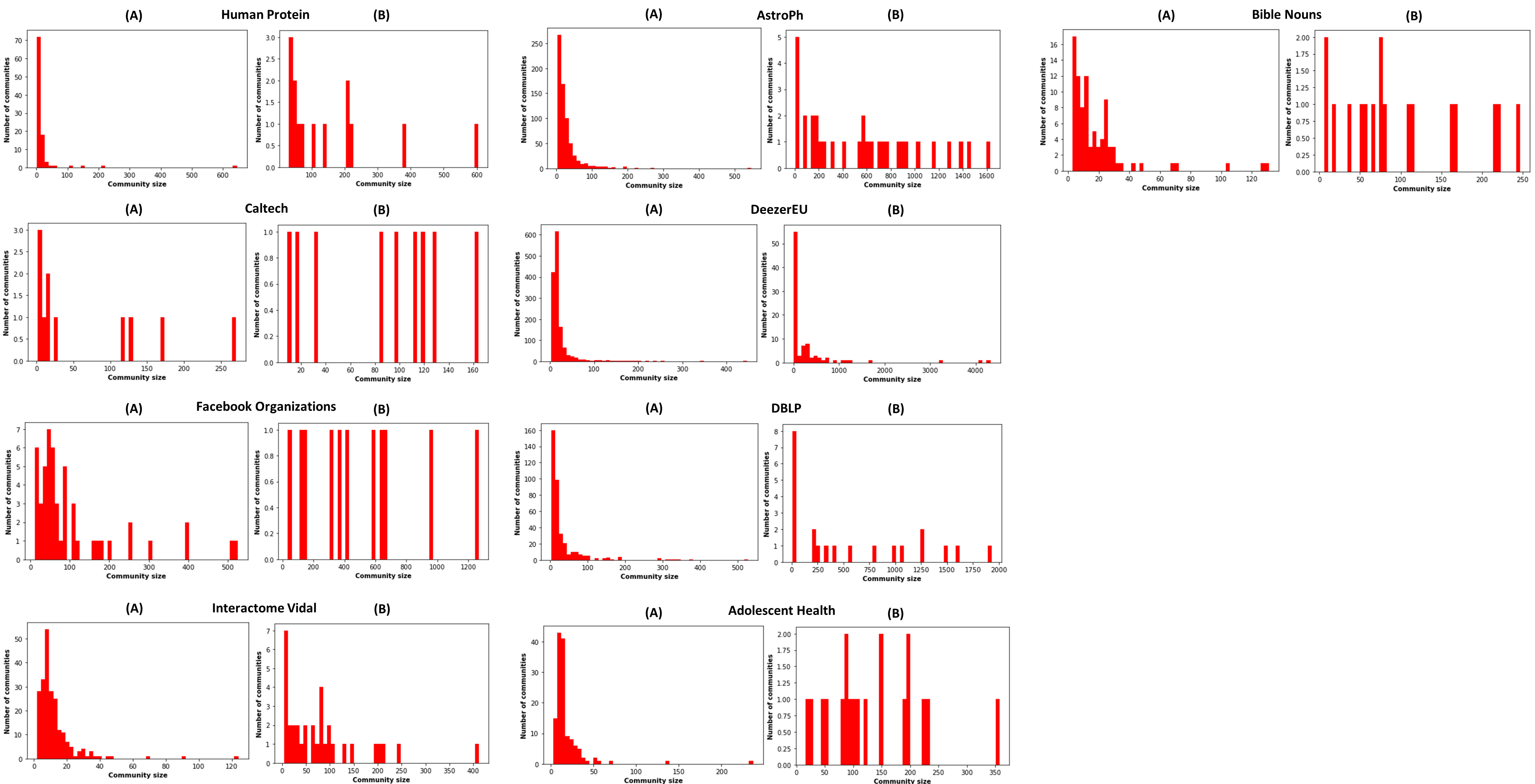

Supplement: S23 Fig — Communities are identified by the Infomap (A) and Louvain (B) community detection algorithms. (PNG) [file pone.0273610.s023.png]
