# Supplementary material for: Ranking influential nodes in complex networks with community structure
Source: PLoS One. 2022 Aug 29;17(8):e0273610. doi: 10.1371/journal.pone.0273610 (PMC9423620; doi:10.1371/journal.pone.0273610)
Supplement: S1 Text — (DOCX) [file pone.0273610.s024.docx]

Ranking influential nodes in complex networks with community structure

Stephany Rajeh ^1*^ and Hocine Cherifi ^1^

^1^ Laboratoire d’Informatique de Bourgogne, University of Burgundy, Dijon, France.

***Corresponding author**

E-mail: stephany.rajeh@u-bourgogne.fr

**Supplementary Methods**

# Centrality Measures

This work investigates six centrality measures using the community-aware and the descending order ranking schemes. One can categorize them into three groups, namely neighborhood-based (Degree and Maximum Neighborhood Component), path-based (Betweenness and Closeness), and iterative refinement-based (Katz and PageRank).

Let $G(V,E)$ be a simple graph where $V$ is the set of nodes $E\subseteq V\times V$ is the set of edges, and $N=|V|$ is the total number of nodes in the network. Let the adjacency matrix $A=a(i,j)$ denote the connectivity between the nodes $i$ and $j$ where $a\left( i,j \right)=1$ if both nodes are connected and $a\left( i,j \right)=0$, otherwise. The adjacency matrix can also be weighted in case networks are weighted by substituting the binary connections with weights. Let the neighborhood of node $i$ be defined as the set $\mathbb{N}_{p}(i)=\{j\in V:\left( i,j \right)\in E\}$ at length $p$, where $p=1,2,\ldots, D$ such that $D$ is the diameter of $G.$ Consequently, two nodes are neighbors of order $A^{p}$ if there is a minimal path connecting the two nodes at $p$ steps. Finally, let us consider $\alpha(i)$ as a measure of the centrality of node $i$ in graph $G$. In the subsequent sections, we introduce the definitions of the centrality measures.

## Neighborhood-based Centrality Measures

### Degree Centrality

The Degree centrality of a node sums the total number of connections a node has in its direct neighborhood. It can be defined as:

$$\alpha_{d}(i)=\sum_{j=1}^{N} \alpha(i,j)$$

where $\alpha(i,j)$ is based on $A^{p=1}$.

### Maximum Neighborhood Component Centrality

The Maximum Neighborhood Component centrality extracts the largest connected component from the direct neighborhood of a node to quantify its importance. It can be defined as:

$$\alpha_{m}(i)=|LCC\in\mathbb{N}_{1}(i)|$$

where $\mathbb{N}_{1}(i)$ is the set of direct neighbors of node $i$.

## Path-based Centrality Measures

### Betweenness Centrality

Betweenness centrality is based on the frequency a node situated in the shortest path between any other two nodes in the network. It is defined as:

$$\alpha_{b}\left( i \right)=\sum_{s,t\neq i} \frac{\sigma_{i}\left( s,t \right)}{\sigma(s,t)}$$

where $\sigma(s,t)$ quantifies the frequency of shortest paths between nodes $s$ and $t$ and $\sigma_{i}(s,t)$ quantifies the frequency of shortest paths between nodes $s$ and $t$ such that node $i$ is present in these paths too.

### Closeness Centrality

Closeness centrality is based on the inverse distance or how close it is to all other nodes in the network. It is defined as:

$$\alpha_{c}\left( i \right)=\frac{N-1}{\sum_{j=1}^{N} d(i,j)}$$

where $d(i,j)$ is the distance between nodes $i$ and $j$.

## Iterative Refinement-based Centrality Measures

### Katz Centrality

Katz centrality quantifies a node's importance based on its connections to all the nodes in the network. However, distant nodes are penalized by an attenuation parameter. It is defined as:

$$\alpha_{k}\left( i \right)=\sum_{p=1}^{\infty} \sum_{j=1}^{N} \alpha^{p}\left( i,j \right)s^{p}$$

where $\alpha^{p}\left( i,j \right)$ denotes the connectivity of node $i$ to all nodes $j$ at distance $p$ and $s^{p}$ is the attenuation factor such that it is less than the reciprocal of the absolute value of the adjacency matrix $A's$ largest eigenvalue.

### PageRank Centrality

PageRank centrality is based on the quantity and quality of the node's direct and indirect connections. It can be thought of as a Markov chain process. It is defined as:

$$\alpha_{p}\left( i \right)=\frac{1-\delta}{N}+\delta\sum_{j\in\mathbb{N}_{1}} \frac{\alpha_{p}\left( j \right)}{k_{j}}$$

where $\alpha_{p}\left( i \right)$ and $\alpha_{p}\left( j \right)$ are the PageRank centralities of nodes $i$ and $j$, respectively, $\mathbb{N}_{1}$is the set of direct neighbors of node $i$, $k_{j}$ is the number of links from node $j$ to node $i$, and $\delta$ is the damping parameter such that $d\in[0,1]$.

# Susceptible-Infected-Recovered (SIR) Model

The Susceptible-Infected-Recovered (SIR) model is a widely used diffusion model in networks [3]. Indeed, it can simulate many real-world diffusion phenomena such as diseases, awareness campaigns, cyberattacks, and viral marketing. At the start of the diffusion process, all nodes are in the susceptible state $(S)$ except for a set of nodes $(f_{o})$ which are set in the infectious state ($I)$. To compare the diffusion power of any centrality measure, the set of nodes initially infected is chosen according to the latter's ranking (usually in the descending order). At each step, any node in the infectious state infects its neighbors in the susceptible state given a rate $\lambda.$ Concurrently, infected nodes can be recovered and cannot infect others anymore at a rate $\gamma$. The process stops when nodes in the infectious state are inexistent. Being left with nodes in the susceptible and recovered states, one infers from the nodes recovered the diffusion power of the initial set of nodes $f_{o}$ ranked according to a centrality measure. Usually called the “outbreak size”, the centrality measures’ goal is to maximize this value. In our study, a SIR model experiment involves 100 independent simulations. For a given centrality measure, we use the spreading outbreak of the descending order ranking scheme as a baseline to compare it with the community-aware ranking scheme. Accordingly, the relative difference in the outbreak size is defined as:

$$\Delta R=\frac{R_{c}-R_{b}}{R_{b}}$$

where $R_{c}$ is the outbreak size based on the community-aware ranking scheme of any centrality measure and $R_{b}$ is the outbreak size based on the descending order ranking scheme of the same centrality measure. If $\Delta R$ is positive, the community-aware ranking scheme is more effective than the descending order ranking scheme. Otherwise, $\Delta R$ is negative.

Note that since each network has an epidemic threshold $(\lambda_{th})$ which controls the spreading outbreak. We set it slightly higher than the threshold, which is quantified as the following [2]:

$$\lambda_{th}=\frac{<k>}{<k^{2}>-<k>}$$

where $<k>$ and $<k^{2}>$ are the first and second moments of the network’s degree distribution.

# Community Detection Algorithms

In real-world networks, if the community structure is not known a priori, a community detection is needed to uncover it. In this study, we use two fundamentally different community detection algorithms to examine the robustness of the community-aware ranking scheme.

## Infomap

The Infomap algorithm [3] exploits the concept of data compression for a random walker. Since a modular structure characterizes most real-world networks, random walkers tend to stay longer inside communities. Codewords can then be used as prefixes for each community. Also, codewords for each node in a given community are assigned (which can be reused across different communities). When the random walker is inside the same community, prefix codes used are minimal. Jumping from one community to another, prefix codes increase. The goal is to build a code that detects communities so that the random walker’s shortest description is acquired.

## Louvain

The Louvain algorithm [4] is based on optimizing the modularity of a network. It consists of two iterative steps. First, communities are uncovered by maximizing local modularity between the nodes. Second, a new network is built. It consists of nodes of the communities found in the previous step. The algorithm keeps running to maximize modularity on the new network until no further gain is possible.

**Supplementary Results**

**Table 1. The Degree centrality of each node in the toy network with their respective descending order and community-aware ranks.**

| Node ID | Degree | Descending order ranks | Community-aware ranks |
| --- | --- | --- | --- |
| 1 | 5 | 2 | 4 |
| 2 | 3 | 12 | 16 |
| 3 | 3 | 13 | 18 |
| 4 | 5 | 3 | 7 |
| 5 | 7 | 1 | 1 |
| 6 | 4 | 4 | 10 |
| 7 | 4 | 5 | 13 |
| 8 | 2 | 17 | 20 |
| 9 | 2 | 18 | 21 |
| 10 | 2 | 19 | 22 |
| 11 | 2 | 20 | 15 |
| 12 | 3 | 14 | 12 |
| 13 | 4 | 6 | 3 |
| 14 | 4 | 7 | 6 |
| 15 | 4 | 8 | 9 |
| 16 | 2 | 21 | 17 |
| 17 | 3 | 15 | 11 |
| 18 | 4 | 9 | 5 |
| 19 | 4 | 10 | 2 |
| 20 | 3 | 16 | 14 |
| 21 | 4 | 11 | 8 |
| 22 | 2 | 22 | 19 |

**Table 2. The Betweenness centrality of each node in the toy network with their respective descending order and community-aware ranks.**

| Node ID | Betweenness | Descending order ranks | Community-aware ranks |
| --- | --- | --- | --- |
| 1 | 0.039 | 14 | 18 |
| 2 | 0.056 | 13 | 16 |
| 3 | 0.200 | 5 | 1 |
| 4 | 0.099 | 11 | 13 |
| 5 | 0.158 | 7 | 4 |
| 6 | 0.100 | 10 | 10 |
| 7 | 0.146 | 8 | 7 |
| 8 | 0.010 | 19 | 21 |
| 9 | 0.014 | 17 | 20 |
| 10 | 0.000 | 20 | 22 |
| 11 | 0.016 | 16 | 15 |
| 12 | 0.038 | 15 | 12 |
| 13 | 0.287 | 1 | 3 |
| 14 | 0.266 | 3 | 9 |
| 15 | 0.280 | 2 | 6 |
| 16 | 0.000 | 21 | 17 |
| 17 | 0.012 | 18 | 14 |
| 18 | 0.080 | 12 | 11 |
| 19 | 0.125 | 9 | 8 |
| 20 | 0.190 | 6 | 5 |
| 21 | 0.221 | 4 | 2 |
| 22 | 0.000 | 22 | 19 |

**Table 3. The number of communities and their minimum and maximum sizes for the LFR synthetic networks generated with different community size distribution exponents. Networks are sorted from strong to weak community structure strengths while keeping other parameters fixed.**

| Network $\theta=2$ | Number of communities | Minimum Size | Maximum Size |
| --- | --- | --- | --- |
| $\mu=0.05$ | 145 | 5 | 224 |
| $\mu=0.10$ | 119 | 6 | 200 |
| $\mu=0.20$ | 125 | 6 | 163 |
| $\mu=0.40$ | 183 | 4 | 230 |
| $\mu=0.70$ | 124 | 4 | 231 |
| Network $\theta=3$ | Number of communities | Minimum Size | Maximum Size |
| $\mu=0.05$ | 251 | 6 | 63 |
| $\mu=0.10$ | 255 | 5 | 134 |
| $\mu=0.20$ | 268 | 5 | 38 |
| $\mu=0.40$ | 353 | 4 | 62 |
| $\mu=0.70$ | 344 | 4 | 104 |

**Table 4. Topological characteristics of real-world networks based on Infomap.** $N$ **is the total number of nodes.** $|E|$ **is the total number of edges.** $\mu$ **is the mixing parameter.** $Q$ **is the modularity.** $ϰ$ **is the average internal degree.** $\varphi$ **is the average internal distance.** $\varphi$ **is the average internal density.**

| Network ($N;\vert E\vert$) | $\mu$ | $Q$ | $ϰ$ | $\varphi$ | $\psi$ |
| --- | --- | --- | --- | --- | --- |
| EU Airlines (417; 2,953) [5] | 0.07 | 0.11 | 3.97 | 1.49 | 0.60 |
| Ego Facebook (4,039; 88,234) [6] | 0.08 | 0.81 | 14.69 | 1.53 | 0.54 |
| U.S. Airports (500; 2,980) [7] | 0.08 | 0.16 | 2.30 | 1.37 | 0.70 |
| Facebook Friends (329; 1,984) [5] | 0.11 | 0.69 | 5.17 | 1.54 | 0.55 |
| Facebook Pol. (5,908; 41,729) [6] | 0.11 | 0.84 | 5.33 | 1.85 | 0.40 |
| Madrid Train Bomb. (64; 243) [7] | 0.12 | 0.31 | 4.25 | 1.59 | 0.53 |
| Yeast Collins (1,004; 8,319) [5] | 0.12 | 0.75 | 6.99 | 1.52 | 0.62 |
| Malaria Genes (307; 2,812) [5] | 0.13 | 0.63 | 10.42 | 1.47 | 0.60 |
| NetSci (379; 914) [6] | 0.14 | 0.81 | 3.69 | 1.53 | 0.54 |
| Reptiles (496; 984) [6] | 0.15 | 0.81 | 2.77 | 1.85 | 0.44 |
| Marvel Partnerships (181; 224) [5] | 0.15 | 0.81 | 2.01 | 1.86 | 0.41 |
| 911AllWords (13,308; 148,035) [8] | 0.16 | 0.05 | 1.72 | 1.63 | 0.60 |
| U.S. Power Grid (4,941; 6,594) [7] | 0.16 | 0.83 | 2.08 | 2.56 | 0.24 |
| Board of Directors (854; 2,745) [5] | 0.16 | 0.82 | 5.16 | 1.49 | 0.58 |
| PGP (10,680; 24,316) [8] | 0.17 | 0.81 | 2.40 | 1.97 | 0.39 |
| Princeton (6,575; 293,307) [6] | 0.19 | 0.33 | 15.74 | 1.77 | 0.48 |
| London Transport (369; 430) [5] | 0.20 | 0.78 | 1.83 | 2.49 | 0.31 |
| EuroRoad (1,039; 1,305) [6] | 0.20 | 0.79 | 1.94 | 2.70 | 0.25 |
| Internet Top. Cog. (197; 243) [5] | 0.20 | 0.75 | 1.91 | 2.23 | 0.33 |
| DNC Emails (849; 10,384) [5] | 0.20 | 0.42 | 5.70 | 1.40 | 0.63 |
| Yeast Protein (1,458; 1,993) [6] | 0.24 | 0.75 | 1.94 | 2.04 | 0.32 |
| Blumenau Drug (75; 181) [5] | 0.28 | 0.69 | 1.69 | 1.66 | 0.47 |
| Retweets Copen. (761; 1,029) [6] | 0.29 | 0.70 | 1.80 | 1.93 | 0.34 |
| Hamsterster (1,788; 12,476) [7] | 0.29 | 0.39 | 3.52 | 1.86 | 0.37 |
| Human Protein (1,788; 12,476) [7] | 0.35 | 0.47 | 2.03 | 2.06 | 0.25 |
| Caltech (762; 16,651) [6] | 0.36 | 0.39 | 13.35 | 1.79 | 0.43 |
| Facebook Org. (5,524; 94,219) [5] | 0.37 | 0.59 | 15.09 | 1.95 | 0.27 |
| Interactome Vidal (5,524; 94,219) [5] | 0.39 | 0.58 | 2.08 | 2.20 | 0.30 |
| AstroPh (5,524; 94,219) [6] | 0.42 | 0.56 | 6.62 | 1.89 | 0.41 |
| DeezerEU (28,281; 92,752) [9] | 0.43 | 0.57 | 2.59 | 2.48 | 0.22 |
| DBLP (12,494; 49,579) [5] | 0.43 | 0.55 | 2.54 | 2.26 | 0.20 |
| Adol. Health (2,539; 10,455) [7] | 0.44 | 0.57 | 2.29 | 0.28 | 0.70 |
| Bible Nouns (1,707; 9,059) [5] | 0.50 | 0.46 | 4.00 | 1.88 | 0.36 |

**Table 5. The number of communities and their minimum and maximum sizes for the real-world networks based on communities identified by Infomap.**

| Network | Number of communities | Minimum Size | Maximum Size |
| --- | --- | --- | --- |
| EU Airlines | 10 | 2 | 332 |
| Ego Facebook | 72 | 2 | 471 |
| U.S. Airports | 39 | 2 | 226 |
| Facebook Friends | 21 | 2 | 72 |
| Facebook Politician Pages | 180 | 2 | 421 |
| Madrid Train Bombings | 5 | 3 | 38 |
| Yeast Collins | 61 | 2 | 119 |
| Malaria Genes | 11 | 2 | 86 |
| NetSci | 38 | 3 | 32 |
| Reptiles | 55 | 2 | 42 |
| Marvel Partnerships | 27 | 2 | 11 |
| 911AllWords | 842 | 2 | 7609 |
| U.S. Power Grid | 422 | 3 | 44 |
| Board of Directors | 78 | 5 | 23 |
| PGP | 896 | 2 | 160 |
| Princeton | 29 | 2 | 3714 |
| London Transport | 50 | 4 | 14 |
| EuroRoad | 111 | 3 | 22 |
| Internet Topology Cogentco | 27 | 4 | 19 |
| DNC Emails | 38 | 2 | 231 |
| Yeast Protein | 164 | 2 | 49 |
| Blumenau Drug | 6 | 3 | 29 |
| Retweets Copenhagen | 92 | 3 | 32 |
| Hamsterster | 64 | 2 | 692 |
| Human Protein | 99 | 2 | 645 |
| Caltech | 11 | 2 | 270 |
| Facebook Organizations | 51 | 11 | 526 |
| Interactome Vidal | 222 | 2 | 124 |
| AstroPh | 675 | 2 | 547 |
| DeezerEU | 1395 | 2 | 446 |
| DBLP | 376 | 2 | 528 |
| Adolescent Health | 136 | 3 | 237 |
| Bible Nouns | 88 | 3 | 131 |

**Table 6. The number of communities and their minimum and maximum sizes for the real-world networks based on communities identified by Louvain.**

| Network | Number of communities | Minimum Size | Maximum Size |
| --- | --- | --- | --- |
| EU Airlines | 8 | 27 | 103 |
| Ego Facebook | 15 | 19 | 548 |
| U.S. Airports | 12 | 2 | 137 |
| Facebook Friends | 10 | 5 | 92 |
| Facebook Politician Pages | 29 | 17 | 585 |
| Madrid Train Bombings | 5 | 3 | 22 |
| Yeast Collins | 22 | 3 | 130 |
| Malaria Genes | 8 | 6 | 71 |
| NetSci | 18 | 6 | 56 |
| Reptiles | 19 | 6 | 55 |
| Marvel Partnerships | 14 | 6 | 19 |
| 911AllWords | 14 | 5 | 2052 |
| U.S. Power Grid | 41 | 26 | 241 |
| Board of Directors | 26 | 13 | 69 |
| PGP | 101 | 6 | 672 |
| Princeton | 11 | 4 | 1752 |
| London Transport | 17 | 11 | 31 |
| EuroRoad | 23 | 20 | 84 |
| Internet Topology Cogentco | 12 | 6 | 27 |
| DNC Emails | 10 | 2 | 210 |
| Yeast Protein | 33 | 9 | 84 |
| Blumenau Drug | 5 | 12 | 19 |
| Retweets Copenhagen | 23 | 7 | 85 |
| Hamsterster | 13 | 6 | 307 |
| Human Protein | 14 | 32 | 604 |
| Caltech | 9 | 9 | 164 |
| Facebook Organizations | 11 | 35 | 1267 |
| Interactome Vidal | 34 | 4 | 412 |
| AstroPh | 32 | 5 | 1629 |
| DeezerEU | 91 | 4 | 4326 |
| DBLP | 22 | 5 | 1933 |
| Adolescent Health | 19 | 16 | 358 |
| Bible Nouns | 17 | 6 | 247 |

**Supplementary References**

1. Anderson, Roy M., and Robert M. May. "Population biology of infectious diseases: Part I." Nature 280.5721 (1979): 361-367.

2. Wang, Wei, et al. "Predicting the epidemic threshold of the susceptible-infected-recovered model." Scientific reports 6.1 (2016): 1-12.

3. Rosvall, M. & Bergstrom, C. T. Maps of random walks on complex networks reveal community structure. Proc. Natl. Acad. Sci. 105, 1118–1123 (2008)

4. Blondel, V. D., Guillaume, J.-L., Lambiotte, R. & Lefebvre, E. Fast unfolding of communities in large networks. J. statistical mechanics: theory experiment 2008, P10008 (2008).

5. Peixoto, T. P. "The netzschleuder network catalogue and repository (2020)." URL https://networks. skewed. de (2020).

6. Rossi, R., & Ahmed, N. (2015, March). The network data repository with interactive graph analytics and visualization. In Twenty-ninth AAAI conference on artificial intelligence.

7. Kunegis, Jérôme. "Handbook of Network Analysis [KONECT--the Koblenz Network Collection]." arXiv preprint arXiv:1402.5500 (2014).

8. Clauset, Aaron, Ellen Tucker, and Matthias Sainz. "The Colorado index of complex networks." Retrieved July 20.2018 (2016): 22.

9. Rozemberczki, B., & Sarkar, R. (2020, October). Characteristic functions on graphs: Birds of a feather, from statistical descriptors to parametric models. In Proceedings of the 29th ACM international conference on information & knowledge management (pp. 1325-1334).
